# Chalcogen-Varied Imidazolone Derivatives as Antibiotic Resistance Breakers in *Staphylococcus aureus* Strains

**DOI:** 10.3390/antibiotics12111618

**Published:** 2023-11-11

**Authors:** Karolina Witek, Aneta Kaczor, Ewa Żesławska, Sabina Podlewska, Małgorzata Anna Marć, Kinga Czarnota-Łydka, Wojciech Nitek, Gniewomir Latacz, Waldemar Tejchman, Markus Bischoff, Claus Jacob, Jadwiga Handzlik

**Affiliations:** 1 Department of Technology and Biotechnology of Drugs, Jagiellonian University Medical College, Medyczna 9, 30-688 Krakow, Poland; karolina.witek@uj.edu.pl (K.W.); aneta.kaczor@uj.edu.pl (A.K.); smusz@if-pan.krakow.pl (S.P.); marcmalgorzata@gmail.com (M.A.M.); kinga.czarnota@doctoral.uj.edu.pl (K.C.-Ł.); glatacz@cm-uj.krakow.pl (G.L.); 2 Department of Pharmaceutical Microbiology, Jagiellonian University Medical College, Medyczna 9, 30-688 Krakow, Poland; 3 Bioorganic Chemistry, School of Pharmacy, University of Saarland, Campus B2.1, D-66123 Saarbrüecken, Germany; c.jacob@mx.uni-saarland.de; 4 Institute of Medical Microbiology and Hygiene, Saarland University, D-66421 Homburg, Germany; markus.bischoff@uniklinikum-saarland.de; 5 Institute of Biology and Earth Sciences, Pedagogical University of Krakow, Podchorążych 2, 30-084 Krakow, Poland; ewa.zeslawska@up.krakow.pl (E.Ż.); waldemar.tejchman@up.krakow.pl (W.T.); 6 Maj Institute of Pharmacology, Polish Academy of Sciences, Smętna 12, 31-343 Krakow, Poland; 7 Doctoral School of Medical and Health Sciences, Jagiellonian University Medical College, Św. Łazarza 15, 31-530 Krakow, Poland; 8 Faculty of Chemistry, Jagiellonian University, Gronostajowa 2, 30-387 Krakow, Poland; nitek@chemia.uj.edu.pl

**Keywords:** *Staphylococcus aureus*, MRSA, 5-arylideneimidazoline-4-ones, thiazole(s), imidazolones, hydantoins, antibiotic adjuvant, PBP2a

## Abstract

In this study, a search for new therapeutic agents that may improve the antibacterial activity of conventional antibiotics and help to successfully overcome methicillin-resistant *Staphylococcus aureus* (MRSA) infections has been conducted. The purpose of this work was to extend the scope of our preliminary studies and to evaluate the adjuvant potency of new derivatives in a set of *S. aureus* clinical isolates. The study confirmed the high efficacy of piperazine derivatives of 5-arylideneimidazol-4-one (**7**–**9**) tested previously, and it enabled the authors to identify even more efficient modulators of bacterial resistance among new analogs. The greatest capacity to enhance oxacillin activity was determined for 1-benzhydrylpiperazine 5-spirofluorenehydantoin derivative (**13**) which, at concentrations as low as 0.0625 mM, restores the effectiveness of β-lactam antibiotics against MRSA strains. In silico studies showed that the probable mechanism of action of **13** is related to the binding of the molecule with the allosteric site of PBP2a. Interestingly, thiazole derivatives tested were shown to act as both oxacillin and erythromycin conjugators in *S. aureus* isolates, suggesting a complex mode of action (i.e., influence on the Msr(A) efflux pump). This high enhancer activity indicates the high potential of imidazolones to become commercially available antibiotic adjuvants.

## 1. Introduction

*S. aureus* is a Gram-positive bacterium able to cause a wide spectrum of human diseases. It is predominantly responsible for minor skin and soft-tissue infections, but also more serious invasive syndromes such as pneumonia, bacteremia, severe sepsis, and endocarditis [1,2]. MRSA accounts for 20–80% of all nosocomial *S. aureus* infections and poses an increasing challenge for healthcare practitioners [3]. Since the bacterium acquires resistance to practically all antimicrobials introduced into clinical use, including last-resort antibiotics such as daptomycin, vancomycin, and linezolid, diseases caused by MRSA strains are usually difficult to treat. Consequently, due to the limited therapeutic options, the appearance of *S. aureus* in hospital settings has become one of the most serious public health concerns [4,5]. Estimates indicate that the mortality rate from multidrug-resistant *S. aureus* bacteremia, despite improvements in medical care, continues to be 15–50% [6]. To make matters worse, while for a long time MRSA infections were restricted to clinical units, in the last decade MRSA strains have emerged in the community, affecting healthy individuals without any history of hospitalization [3]. The major mechanisms by which *S. aureus* becomes tolerant to β-lactams primarily stem from (i) the production of β-lactamases capable of hydrolyzing and inactivating β-lactam antibiotics before they reach their target, and (ii) the acquisition of the *mecA* gene which encodes modified penicillin-binding proteins (PBP2a) with a lower affinity to β-lactams compared to native *S. aureus* PBPs [7,8,9]. Native PBPs belong to a group of enzymes that are anchored on the bacterial membrane and mediate the formation of peptide cross-links between peptidoglycan chains during the final stage of bacterial cell wall biosynthesis [10,11,12]. The binding of β-lactam antibiotics to PBPs inactivates the function of these enzymes, which ultimately leads to the disruption of proper cross-linking of the peptidoglycan layer, loss of cell wall integrity, and bacterial death [12,13]. Due to the resistance determinant, MRSA overcomes the antibacterial action of β-lactams, since PBP2a provides a normal synthesis of bacterial cell walls whilst susceptible PBPs are otherwise inactivated by the antibiotic [14].

The transcription of *mecA* is regulated by the *mecR1-mecI-mecR2* system, which encode a sensor-inducer, transcriptional repressor, and anti-repressor, respectively. Additionally, transcriptional regulation of *mecA* is also accomplished by the *blaI-blaR1* system, which is homologous to the *mec* gene complex and controls the expression of the *blaZ* gene responsible for both PBP2a and β-lactamase production in MRSA [15,16,17]. Remarkably, it has been shown that bla regulators, if inactivated by genetic or external molecule inhibition, have the capacity to assume the function of the mecR1-mecI-mecR2 system and efficiently control the mecA transcription. [8,18,19].

Regarding this relatively rapid acquisition of antibiotic resistance by MRSA and its expeditious spread throughout the world, a continuous search for alternative methods to ensure effective anti-MRSA treatment is highly required. One of the most promising strategies in this respect is the discovery and development of compounds that potentiate the antimicrobial activity of existing antibiotics against multidrug-resistant bacteria. Since PBP2a play an essential role in the acquisition of β-lactam resistance in staphylococci, inactivation of these proteins represents a significant approach that may be exploited to develop efficacious new agents that, in combination with β-lactams, can rejuvenate the antibacterial activity of the latter against the highly problematic MRSA pathogen [8].

**Figure 1:**
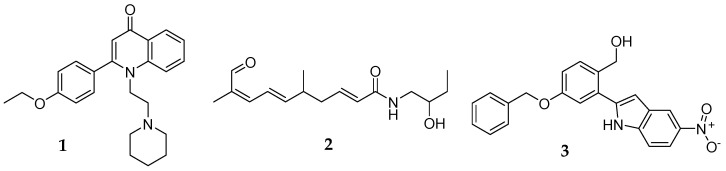
Structures of some potent compounds able to restore antibiotic efficiency (**1**−**3**) [20].

Different groups of compounds have been identified as potent modulators of MRSA resistance to antibiotics, including quinolone, citral amide, chalcones, alkenamides, and indoles derivatives (**1**–**3**, Figure 1) [20].

**Figure 2:**
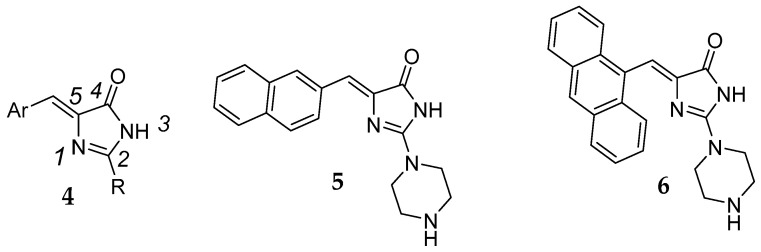
General structure (**4**) and the most active 5-arylidenoimidazolones (**5**,**6**) found previously [21,22].

In our previous research, some 5-arylideneimidazolones with piperazine at position two [20] (Figure 2) or three [21,22] were able to improve the antibacterial activity of β-lactams and fluoroquinolone antibiotic ciprofloxacin against an *S. aureus* bacterium that was resistant to these drugs. Nevertheless, the probable mechanism of action of these compounds was not determined and is probably complex, involving more than one MDR protein target [20]. The following study enabled us to extend the arsenal of active chemosensitizers for new derivatives, enhancing the efficacy of a representative β-lactam antibiotic oxacillin up to 32-fold against a highly resistant MRSA strain. The results of docking studies and molecular simulations suggested that the most probable mechanism of action of the identified oxacillin adjuvants was the interaction of compounds with the allosteric site of PBP2a and the improvement in the binding of oxacillin to the active site of the protein [21].

Apart from 2- or 3-substituted arylideneimidazolones [21,22,23], active “chemosensitizers” of MDR proteins were found in a wider group of imidazolone-derived compounds, especially those containing a hydantoin (imidazolidine-2,4-dione) scaffold, with an aromatic area extended at positions five [23,24,25] or three [26] and substituted with alkylpiperazines at position one. Although structure–activity relationship analysis for the active imidazolones has not provided either quantitative data or distinct chemical features responsible for these antibiotic adjuvant actions, a bulky hydrophobic/aromatic area at position five of the imidazolone ring was the most common structural trend observed in the majority of the active compounds.

On the other hand, the imidazolone-derived rings, including various exo- or endocyclic chalcogens, i.e., S or Se, are a popular core of active compounds against MDR bacteria [27,28,29]. Furthermore, our previous studies have shown that, in particular, Se-containing compounds displayed great potential in combating bacterial MDR, including that of various MRSA strains [30,31].

Based on the aforementioned previous results indicating the pronounced antibiotic-adjuvant activity of imidazolones containing 5-aromatic substitutions, we selected three chemotypes of structures with that scaffold (Table 1), i.e., 5-arylideneimidazolones (group A), 5-spirofluoreneimidazolones (group B), and S/Se-containing 5-arylideneimidazolone-derived (group C) compounds, in order to expand our investigation by increasing the number of *S. aureus* clinical isolates enrolled in the study and by exploring the chemosensitizing potential of the compounds in conjunction with other classes of antistaphylococcal drugs. In detail, 15 compounds, including methylpiperazine-derived 5-arylideneimidazolones **7**–**10** (group A), a series of 5′-(spiro)fluorenehydantoins with arylpiperazines connected through an alkyl linker to position 1′ and with a variety of small substituents at position 3′ of the hydantoin core **11**–**15** (group B), as well as 5-arylideneimidazolne-derived compounds with varied endo- or exocyclic chalcogen atoms (S, Se) and additional aromatic moieties at positions N3 of the heterocyclic ring (**16**–**21**, group C) were included in the approach (Table 1).

To investigate the potential mechanism of biological action of the series, docking studies to PBP2a, followed by molecular dynamic simulations, were performed. Based on the obtained results, the structure–activity relationship was analyzed. For the most active chemosensitizers found in this study, safety assays in vitro, also supported with in silico simulation, were carried out.

## 2. Results

### 2.1. Chemistry

The investigated compounds (**7**–**21**) were synthesized according to the methods described previously [21,32,33,34,35,36]. The synthesis of compounds **7**–**10** [21], **12**–**15** [33], **16**–**19** [34,35], and **20** [36] was presented elsewhere, while synthetic procedures with the chemical characteristics of compounds **11** [32] and **21** [36], first described in this study, are presented in Supplementary Materials (Table S1). All compounds (**7**–**21**) were provided to biological assays at a purity >95% (suitable results of spectral or elemental analyses are collected in Supplementary Materials, Table S1).

### 2.2. Crystallographic Studies

**Figure 3:**
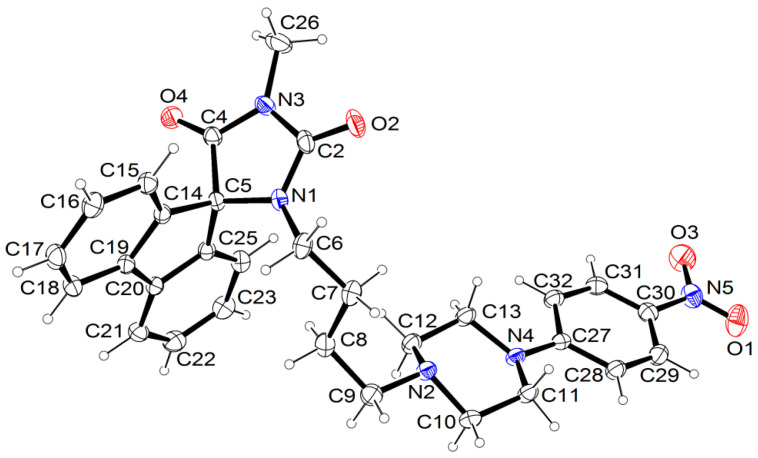
The molecular structure of **12** showing the atom numbering scheme. Displacement ellipsoids are drawn at the 20% probability level.

An overview of the asymmetric units of **12** with the atom numbering are shown in Figure 3.

The hydantoin ring of **12** is perpendicular to the spirofluorene substituent at C5. The angle between the corresponding planes amounts to 88.26(5)°. This arrangement is similar to other 5-spirofluorenehydantoin derivatives with determined crystal structures [32,33,37,38]. The linker between the hydantoin and piperazine rings, consisting of four methylene moieties, is flexible. The torsion angles values being N1-C6-C7-C8 = −76.7(2)°, C6-C7-C8-C9 = −170.4(2)°, and C7-C8-C9-N2 = −56.4(2)° confirm a bent conformation of this linker. The piperazine ring adopts chair conformation with an equatorial p-nitrobenzene substituent at the N4 atom, wherein the hybridization of the N4 atom is closer to sp^2^ than sp^3^. The bond angles at N4 have values of C13-N4-C11 = 114.5(1)°, C11-N4-C27 = 119.5(1)°, and C13-N4-C27 = 118.8(1)°, and the bond length C-N is 1.377(2) Å, which suggests the conjugation of the N4 atom with the aromatic ring.

**Figure 4:**
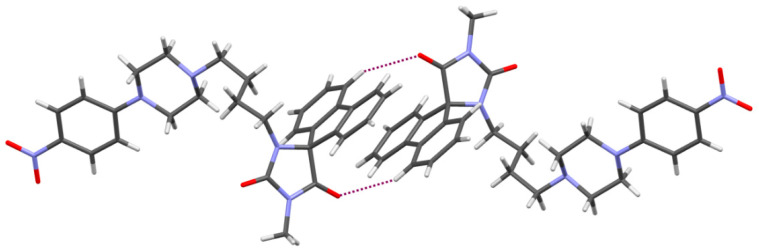
The interactions of two molecules of **12** showing a π-π stacking of spirofluorene substituents. Dashed lines indicate hydrogen bonds.

In the crystal packing, only C-H···O intermolecular interactions are observed, whose parameters are listed in Table 2. The spirofluorene moieties are engaged in π-π interactions (Figure 4) with a distance of 3.5 Å. Similar types of interactions of these moieties are observed in other crystal structures of hydantoin derivatives published earlier [32].

### 2.3. Microbiological Assays

#### 2.3.1. Direct Antibacterial Activity

The results of susceptibility testing performed for compounds **7**–**21** demonstrated that most of the analogs exhibit weak anti-staphylococcal activity (Table S4, Supplementary Materials), thus being good candidates in the search for an adjuvant of antibiotics, helpful to reverse MDR mechanisms. Among the series of compounds investigated, the lowest inhibitory effect was determined for derivative **7**, which, even at a concentration of 1 mM, does not affect the growth of *S. aureus* strains. The MIC values of the remaining compounds are in the range of 0.25–1 mM. It is noteworthy to mention that, for derivatives **16**–**21**, precipitation was observed after addition to a bacterial suspension in MH II broth, therefore, an assessment of the exact MICs of these compounds was not possible. The weak antibacterial activity of imidazolone derivatives allowed us to analyze their chemosensitizing effect in combination with selected antibiotics.

#### 2.3.2. Influence on the Activity of β-Lactam Antibiotics

**Figure 5:**
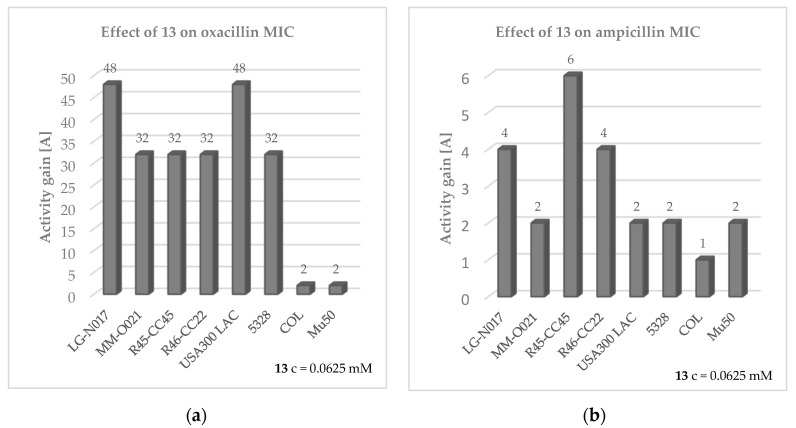
Effect of compound **13** on MIC of antibiotics (**a**) oxacillin and (**b**) ampicillin in MRSA strains. Experiments were performed in 3–4 repetitions. Activity gain (A) was calculated according to Equation (1) (Section 4.2.3), presented as arithmetic mean in case of different MIC values obtained in the repetitions. Compounds were considered as antibiotic potentiators when A ≥ 4.

In the next step of microbiological assays, compounds **7**–**21** were examined for their ability to enhance the antibacterial activity of β-lactam antibiotics against *S. aureus* clinical isolates. For this purpose, either oxacillin or ampicillin was combined with the compounds, tested at the final concentrations corresponding to ¼ of their respective MIC values, or at the highest concentrations at which they do not precipitate and, simultaneously, do not show any antistaphylococcal effect. Oxacillin and ampicillin were chosen due to the relevant difference between these two antibiotics—oxacillin is stable against degradation by most staphylococcal β-lactamases; meanwhile, ampicillin shares the same vulnerability to hydrolytic enzymes as natural penicillin. Accordingly, the reduction of staphylococcal resistance by the compounds tested, when conjointly added to either oxacillin or ampicillin, might provide further insights into the potential mechanism of action of these molecules. Comparison of the effectiveness of antibiotics in the presence and absence of the compounds tested revealed that ten out of fifteen compounds notably improved or even restored the susceptibility of MRSA strains to oxacillin, as determined by the European Committee on Antimicrobial Susceptibility Testing (EUCAST) guidelines (MIC ≤ 2 µg/ml) [39]. Detailed results on the activity of compounds **7**–**21** combined with oxacillin/ampicillin are presented in Supplementary Materials (Table S5–S19). The adjuvant-like effects of the compounds tested in the presence of β-lactams are dose-dependent, with the most pronounced effect observed at the highest concentrations used. The strongest chemosensitizing property was established for compound **13,** which at the concentration of 0.0625 mM reverses oxacillin resistance in six out of eight MRSA strains employed in the study, including LG-NO17, MM-O021, R45-CC45, R46-CC22, USA300 LAC, and 5328. Nevertheless, the compound does not considerably improve oxacillin efficacy in two highly resistant *S. aureus* strains: MRSA COL and vancomycin-intermediate *S. aureus* (VISA) Mu50 (A = 2). Noteworthily, compound **13** at the concentration tested does not have an adjuvant-like property in combination with ampicillin in penicillinase-producing MSSA and MRSA strains. The influence of compound **13** on the effectiveness of oxacillin and ampicillin against *S. aureus* is detailed in Figure 5. 

**Figure 6:**
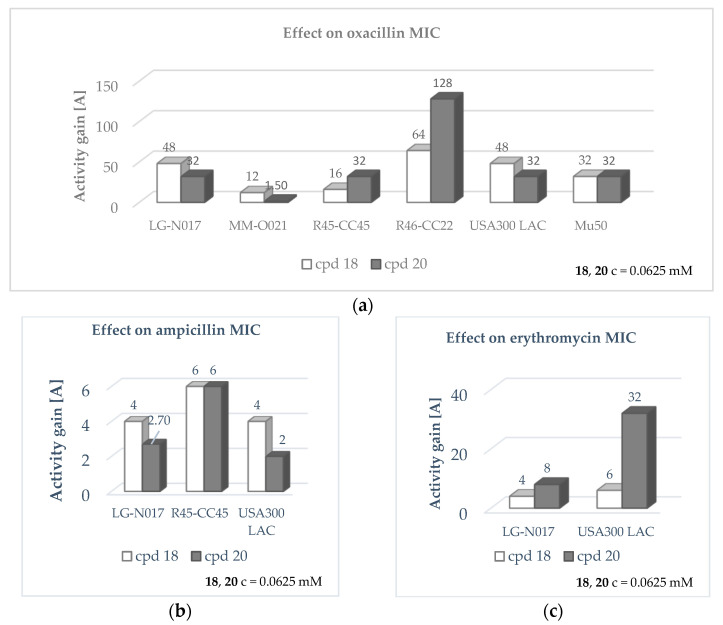
Effect of compounds **18** and **20** on MIC of antibiotics (**a**) oxacillin, (**b**) ampicillin and (**c**) erythromycin in MRSA strains selected for the study.

Similar activity to that described for compound **13** was assessed for the selenium-containing thiazole derivatives **18** and **20**. Compounds at the concentration of 0.03125 mM and 0.0625 mM rejuvenate the antibiotic effect of oxacillin against four MRSA strains comprising the isolate LG-N017, R45-CC45, R46-CC22, and USA300 LAC (Figure 6).

**Figure 7:**
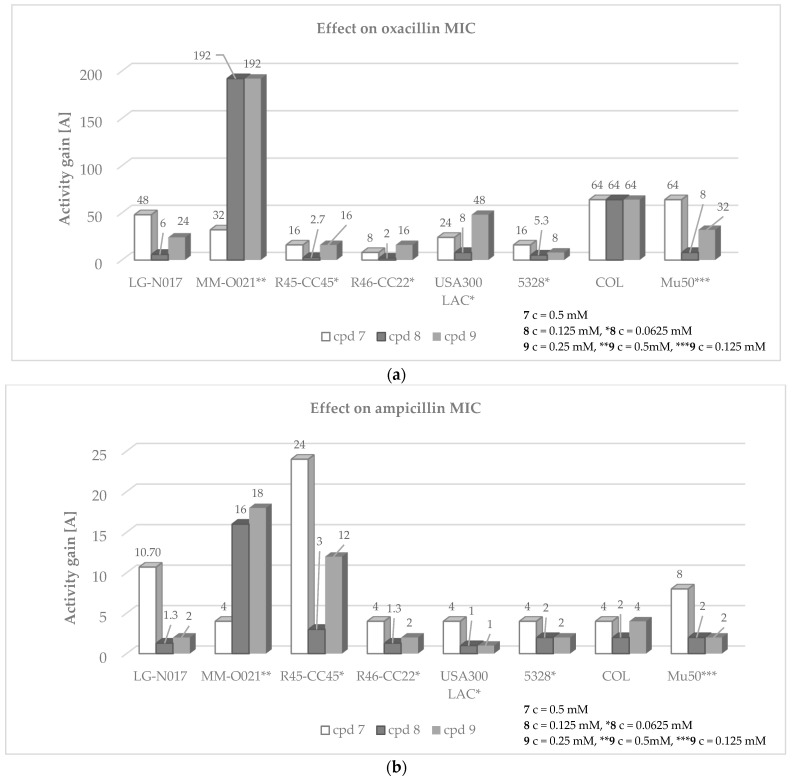
Effect of compounds **7**, **8** and **9** on MIC of (**a**) oxacillin and (**b**) ampicillin in MRSA strains selected for the study.

Pronounced adjuvant activity was also determined for compound **8**, which at the concentration of 0.0625–0.125 mM restores oxacillin activity in six (LG-NO17, MM-O021, R45-CC45, R46-CC22, USA300 LAC, and 5328) MRSA strains (Figure 7). Compound **8** is devoid of potentiating effect in combination with ampicillin against all but one MRSA strain (MM-O021). A similar efficacy to the one determined for **8** was elucidated for derivatives **7** and **9**. Compounds restored the activity of oxacillin against six MRSA strains used in the study; however, the effect was observed at the higher concentrations tested (0.125–0.5 mM). Noteworthily, compound **7**, at a concentration of 0.5 mM, considerably increased the antimicrobial effect of ampicillin towards all *S. aureus* strains (Figure 7). Along with compound **8**, compound **9** had the strongest ability to enhance oxacillin activity against MRSA (Tables S6 and S7, Supplementary Materials). Compounds **8** and **9**, supplemented at a concentration of 0.125 mM and 0.5 mM, respectively, reduce the oxacillin resistance of MM-O021 by 128–256-fold (A = 192). Moreover, both derivatives had one of the strongest adjuvant-like properties in combination with ampicillin in MRSA MM-O021 (A = 16–18). The lowest chemosensitizing effect among fifteen imidazolone derivatives was assessed for compounds **10**, **11**, and **15**, which do not affect oxacillin effectiveness in most of the clinical isolates enrolled in the experiments (Tables S8, S9 and S13, Supplementary Materials). Remarkably, compounds **7**–**21** were devoid of a potentiating effect in the reference MSSA strains MM-O058 and MM-N072. The increase in oxacillin activity in the presence of the compounds tested, and the lack of their impact on ampicillin efficacy, may indicate that the mechanism of anti-MDR action of these molecules may be related to disruption of *mecA* gene expression, regulated by *mecR1-mecI-mecR2* or direct interaction with PBP2a proteins, rather than inhibition of the hydrolyzing activity of β-lactamases. Considering that both *mecR1-mecI-mecR2* and *blaI-blaR1* ensure optimal *mecA* transcription, the adjuvant-like activity of compounds associated with modulation of *mecA* gene by *mec* regulators seems to be less probable.

#### 2.3.3. Influence on the Activity of Macrolide Antibiotic

Next, the ability of compounds **7**–**21** to reduce the MIC of macrolide antibiotics was evaluated. For this purpose, compounds at the same sublethal concentrations as those used in the previous studies were conjointly added to erythromycin and examined for their adjuvant effect. The outcomes obtained in this set of experiments indicated that compounds **18** and **20** enhance erythromycin activity against two MRSA strains resistant to this antibiotic: LG-N017 and USA300 LAC (Figure 5, Table S20 in Supplementary Materials). Notably, combining compound **20** with erythromycin resulted in the restoration of drug susceptibility as determined by the European Committee on Antimicrobial Susceptibility Testing (EUCAST) standard (MIC ≤ 1 µg/mL) for the LG-N017 isolate [39]. Bearing in mind that erythromycin is a well-described substrate of the MsrA efflux pump, these data suggest that the mechanism of the adjuvant action of **18** and **20**, apart from the impact on PBP2a, may be also related to the inhibition of the macrolide transporter expressed by MRSA [40,41].

#### 2.3.4. Influence on Other Classes of Antibiotics

Compounds were also examined for the potential to improve the efficacy of a representative fluoroquinolone antibiotic, ciprofloxacin, and the last-resort glycopeptide drug, vancomycin. Analysis of the results obtained by MIC reduction assay indicated that none of the compounds tested had the ability to considerably increase the effectiveness of either ciprofloxacin against resistant to this antibiotic *S. aureus* Mu50, RR46-CC22 and USA300 LAC strains, or vancomycin against the VISA Mu50 strain (A ≤ 2). 

### 2.4. Molecular Modeling

#### 2.4.1. Docking Studies

The results of molecular modeling studies are analyzed separately for each compound group considered (B, C; the modeling for group A of the compounds has already been published) [21].

**Figure 8:**
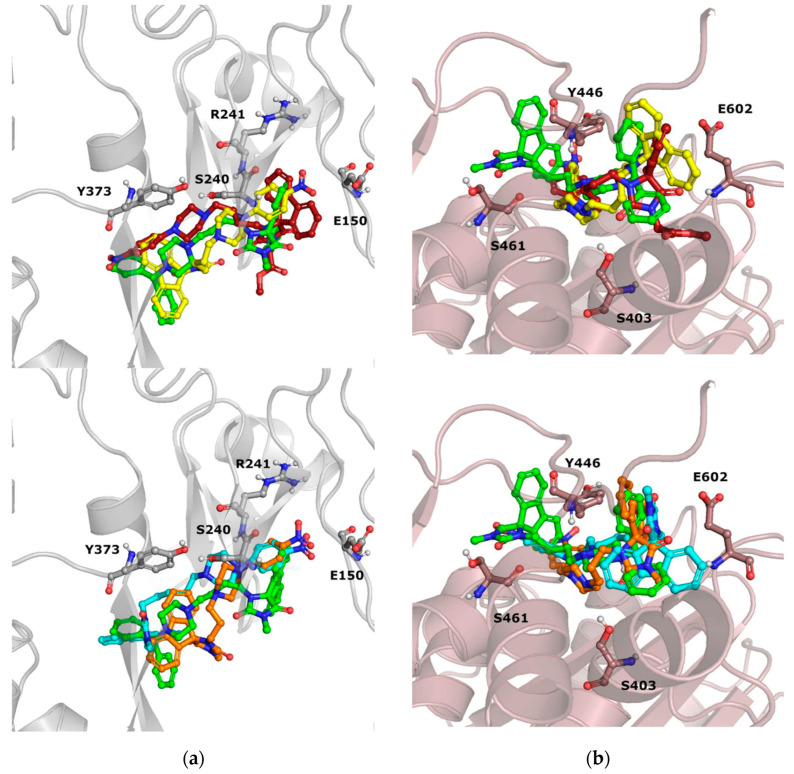
Docking poses obtained for compounds **11**—red, **12**—yellow, **13**—green, **14**—orange, **15**—cyan to (**a**) PBP2a with grid centered on S240, (**b**) PBP2a with grid centered on S403.

Compounds from group B (Figure 8) occupy the same region of the binding sites of PBP2a; however, slight differences in their orientations can be noticed.

Compound **13** was characterized by the highest ability to restore the activity of oxacillin, which is most probably related to the introduction of another phenyl ring at the side of piperazine, instead of the nitro group. The structural modification of **13** in comparison to **11**, **12**, **14**, and **15**, is also related to differences in docking poses of those compounds. Interestingly, in both dockings, to the S240-centered allosteric site and S403 active site, compound **13** adopts a flipped orientation, with the spiro moiety being near the nitro group of **12**, **14**, and **15**. Moreover, in docking to the PBP2a active site, **13** is shifted more towards E460, and forms interactions with E447 and K587. Compound **11**, with an extended R^2^ substituent, adopted a different orientation, which is especially visible in the shifted position of the piperazine moiety.

**Figure 9:**
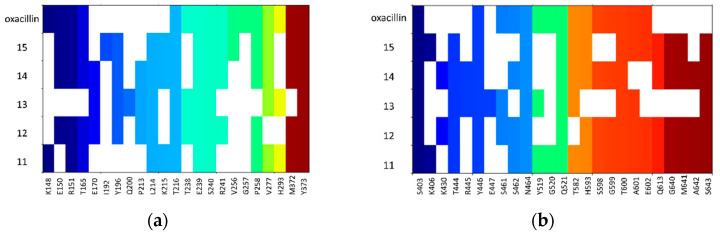
Ligand–Protein Interaction Matrices obtained for compounds from group B (**11**–**15**) and oxacillin for (**a**) S240-docking and (**b**) S403-docking.

Detailed analysis of ligand–protein contacts occurring for compounds **11**–**15** and oxacillin was facilitated thanks to the construction of Ligand–Protein Interaction matrices (Figure 9).

Analysis of the interaction diagrams shows a consistent contact of compounds **11**, **12**, **14**, **15**, and oxacillin with R151, T165, V277, and M372, which is lacking for compound **13**. R151 interacts with the nitro group of a ligand (carboxyl group in the case of oxacillin). On the other hand, only **13** interacts with Q200. Such consistent differences in contacts between **13** and the remaining compounds analyzed are not visible for the analysis of docking to the active site, which is the first premise for the allosteric mechanism of action of **13** (Figure 9b).

**Figure 10:**
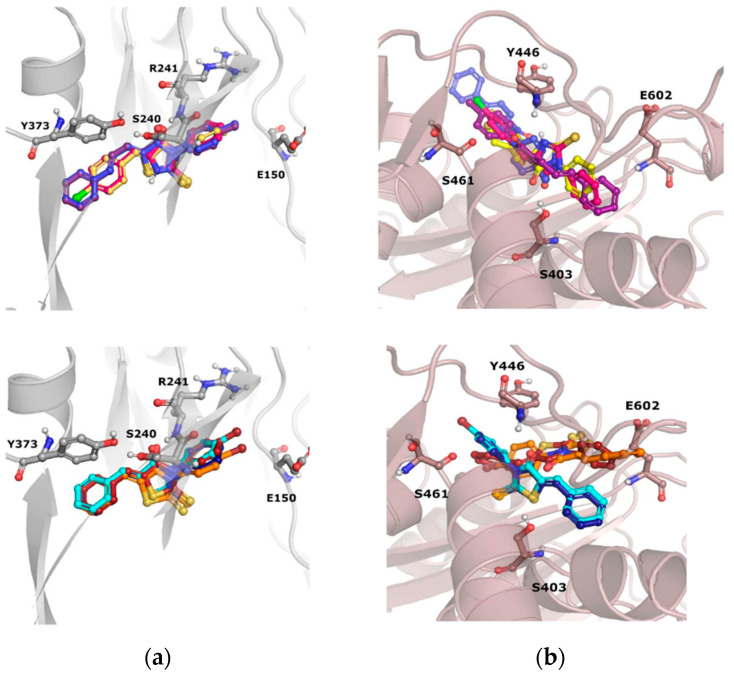
Docking poses obtained for compounds **16**–**21** (**16**—pink, **17**—yellow, **18**—violet, **19**—blue, **20**—dark blue, **21**—orange), docked to (**a**) allosteric site of PBP2a (S240-centered) and (**b**) active site of PBP2a (S403-centered).

In addition, the docking poses of compounds from group C were examined (Figure 10). The orientation of all the compounds from group C (**16**–**21**) is almost the same, when their poses in the PBP2a allosteric site are considered. On the other hand, slight variations in their poses in the PBP2a active site are observed. For example, inactive **19** is shifted towards Y446 in comparison to compounds **16**–**18**. Furthermore, the almost perfectly aligned poses of **20** are different from the orientation of compound **21**. The latter compound adopts a pose in which it is closer to E602 and neighboring amino acids, whereas **20** occupies the PBP2a region, which is closer to S403. Also, almost no differences in the poses of sulfur and selenium counterparts (e.g., **16** and **17**; **18** and **19**) are observed in the PBP2a allosteric site, whereas significant differences occur when docking to the PBP2a active site is considered.

#### 2.4.2. Molecular Dynamic Simulations

**Figure 11:**
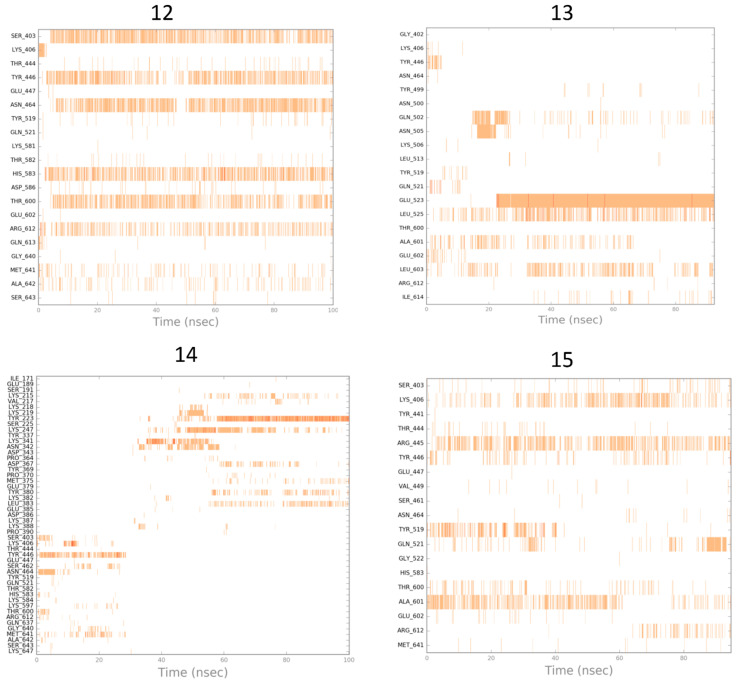
Simulation interaction diagrams obtained for the analyzed compounds during MD simulations (generated for the active site of PBP2a).

**Figure 12:**
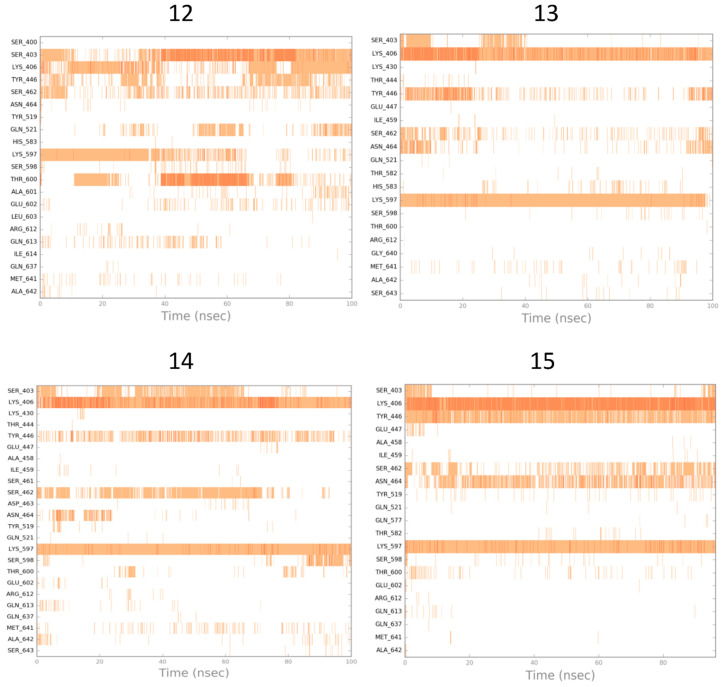
Simulation interaction diagrams obtained for oxacillin during MD simulations (generated for the active site), with the respective compounds present in the allosteric site of PBP2a.

The results of the MD simulation studies carried out for the selected compounds are presented in the form of ligand–protein interaction diagrams generated for the whole simulation time. In order to compare the mechanism of action of the selected compounds, the “stability” of their pose in the binding site was examined (Figure 11); as well, the orientation of oxacillin in the binding site was analyzed when another ligand was present as fitted to the allosteric site of PBP2a (Figure 12).

Changes in ligand–protein interaction in time during MD simulations in Figure 11 suggest that compounds do not rather act via direct interaction with the active site of PBP2a (only **12** was fitted to the active site stably enough, so as action via the PBP2a active site is possible). A much more possible mechanism of compound action is the allosteric modulation of PBP2a, as the oxacillin pose in the active site seems to be unchangeable, with the respective compounds present in the allosteric site.

### 2.5. Structure–Activity Relationship (SAR) Analysis

All compounds (**7**–**21**) tested for the ability to enhance antibiotic efficacy in *S. aureus* strains belong to a family of imidazolone derivatives containing different substituents at positions one, three, and five (group A, B, C) as well as various chalcogens in place of oxygen (S, Se) at position two of the heterocyclic ring (group C). Distinct chemical features of these compounds enabled us to evaluate the influence of certain substituents on the adjuvant-like property within respective groups (A, B, C) of imidazolone derivatives.

Regarding the chemical structures of analogs from group A, the entire population of molecules has two characteristic features, namely, the conservative core of 1H-imidazol-5(4H)-one and the methylpiperazine moiety at position three. In accordance with our earlier findings, the study has shown that the size and kind of aromatic substituents placed within the 5-arylidene fragment of 1H-imidazol-5(4H)-one determined the cooperative action of compounds with the β-lactam antibiotic, oxacillin. Among representative compounds of group A, the most pronounced activity was observed for phenanthrenemethylidene derivative **8**. The compound was able to decrease the effective dose of oxacillin 64- and 128–256-fold in highly resistant MRSA COL and MRSA MM-O021 clinical isolates, respectively. At the same time, **8** had the capacity to rejuvenate the antibacterial activity of oxacillin against six out of eight MRSA strains selected for the study at the lowest concentration tested. Slightly lower anti-MDR potency was identified for compound **9** substituted with the phenoxyphenylmethylidene group at position **5** of the imidazolidine-4-one ring, and this was followed by the activity of its β-naphthylmethylidene analog **7**. The spectrum of activity of **7** and **9** was similar to that determined for **8**; however, the chemosensitizing effect of these compounds was observed at the higher concentrations. Finally, the lowest potency among the three analogs was assessed for anthracenemethylidene derivative **10**, which did not influence oxacillin resistance in most MRSA clinical isolates.

The presence of the 5-spirofluorenehydantoin core and arylpiperazine fragment at position N1 was common for the whole series of molecules from group B. The chemical modifications which seem to be important for adjuvant-like properties of this family of compounds included: (i) alteration of the length of a linker between the hydantoin core and piperazine moiety, (ii) the addition of different kinds of substituents at position one of piperazine and (iii) the introduction of either methyl or ester substituents at position three of the hydantoin ring. Within group B, the strongest capacity to strengthen the antibacterial activity of oxacillin was found in compound **13**, which restored the effectiveness of this antibiotic against six MRSA strains at the lowest concentration among compounds belonging to groups A and B (0.0625 mM). Accordingly, it is reasonable to assume that the 1-benzhydrylpiperazine terminal fragment attached to the hydantoin nucleus by a 4C-long alkyl linker is the best substituent in terms of oxacillin adjuvant activity. Exchanging diphenylmethyl with a *p*-nitrophenyl moiety led to a decrease in the activity, what was observed in the case of **12**. The compound exerted its chemosensitizing potency at a higher dose and was not able to restore oxacillin efficacy against the majority of the MRSA strains. Interestingly, further modifications of the structure of **12** based on the exchange of -CH_3_ with the ester of CH_2_COOCH_3_ (**11**) moiety at the three-position of hydantoin skeleton, as well as the shortening (**14**) or elongation (**15**) of the alkyl linker length, abolished the chemosensitizing activity of the compounds tested. Therefore, the following order of antibiotic adjuvant activity for 5-spirofluorenehydantoin derivatives can be observed: **13** >> **12** > **14** > **11** > **15**.

A high antibiotic adjuvant effect was also found among chalcogen analogs of imidazolone possessing in their structure sulfur and selenium atoms. Two members of this group, namely, *(Z)*-5-((*E*)-3-phenylallylidene)-2-selenoxo-3-*p*-tolylthiazolidin-4-one (**18**) and *(Z)*-5-benzylidene-3-phenyl-2-selenoxothiazolidin-4-one (**20**), at a concentration of 0.03125–0.0625 mM, were able to rejuvenate the efficacy of oxacillin against five and four MRSA clinical isolates, respectively. The SAR analysis performed on the basis of the results obtained indicates that the type of chalcogen substituent attached to the aromatic ring of thiazole plays a crucial role in the cooperative action of compounds with β-lactam antibiotics. It has been demonstrated that the introduction of the selenium atom at position two of the thiazole nucleus determined adjuvant properties of hit compounds **18** and **20**. On the other hand, substituting selenium with a sulfur atom caused a decline in the chemosensitizing effect of the compounds tested. The presence of an oxygen atom at position C4, together with aromatic moieties at positions N3 and C5 of the thiazole core, seems to be another structural feature influencing the capacity of molecules to enhance the efficacy of oxacillin. Remarkably, both selenothiazolidinone derivatives **18** and **20** were also able to improve erythromycin activity in MRSA strains resistant to this antibiotic.

### 2.6. Safety Studies

**Figure 13:**
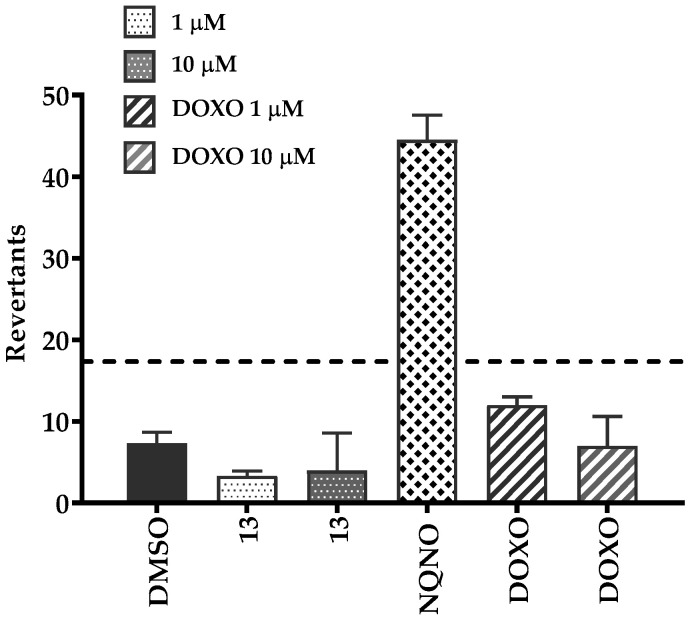
Results of the Ames liquid microtiter test—determination of the mutagenic potential; DMSO—negative control, doxorubicin (DOXO)—reference cytotoxic compound at concentration of 1 µM and 10 µM, NQNO-4-nitroquinoline-*N*-oxide (mutagenic agent); **13**—hydantoin derivative tested at 1 µM and 10 µM concentration, ---- baseline defining the mutagenicity threshold (over this line).

The Ames assay was carried out in accordance with the previously described protocol [42] to check whether the most active compound (**13**) is safe as a non-mutagenic. The MI (mutagenic index) of that compound was below 2.0 at concentrations of 1 µM and 10 µM (Figure 13). Therefore, compound **13** displayed no mutagenicity in both tested concentrations (see details in Table S21 in Supplementary Materials). However, the antibiotic enhancer action of **13** was observed at a concentration of 63 µM, which was too high to be investigated in this AMES test due to condition limitations (prokaryotic model, the manufacturer’s recommended concentration is up to 10 µM).

**Figure 14:**
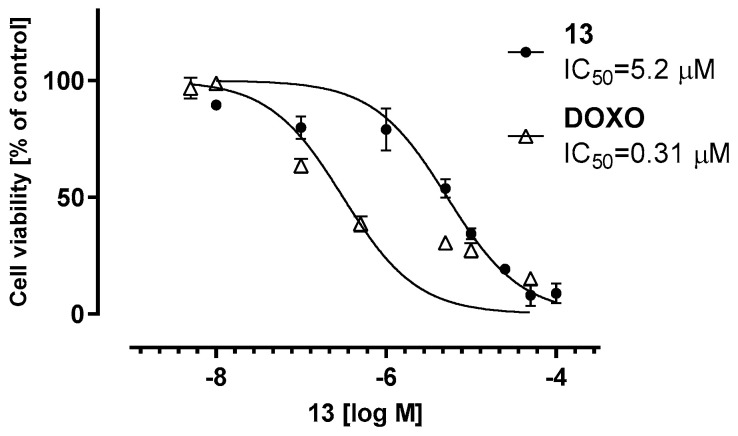
The viability of the HEK-293 cell line after incubation with compound **13** for 72 h. DOXO—doxorubicin. IC_50_ values were calculated by GraphPad Prism 8.0.1 software.

To obtain more information on the safety of compound **13** in eukaryotic cells, the cytotoxicity of the compound was tested using the HEK-293 cell line model in a wide range of concentrations (0.01–100 µM), in comparison to anticancer drug doxorubicin as a positive control. The obtained IC_50_ values (Figure 14) indicate the much weaker cytotoxic effects of **13** compared to doxorubicin. However, the IC_50_ value for **13** was more than 10 times lower than the concentration of its antibiotic “adjuvant” activity.

The results of in vitro safety studies obtained suggest a low risk of mutagenicity, and a visible risk of cytotoxic effects on eukaryotic cells, for compound **13**.

## 3. Discussion

Our previous approach indicated that the mode of action of imidazolone chemosensitizers **7**, **8**, and **9** is most probably related to the allosteric interaction of compounds with PBP2a, which is known as the major determinant of MRSA resistance to β-lactam antibiotics [21]. Considering the low chemosensitizing effect of compounds belonging to group B, in combination with vulnerable to hydrolysis by staphylococcal β-lactamases ampicillin in penicillinase-producing strains, and the fact that they did not exert any activity in the reference MSSA strains, it has been assumed that the activity of new oxacillin adjuvants was also associated with some kind of direct interactions with PBP2a. To address this objective further, docking studies of the binding of compounds **11**–**15** to the crystal structure of PBP2a were carried out. It has been found that compound **13** adopts different docking poses than compounds for which less profound activity was determined (**11**, **12**, **14**, and **15**). Moreover, analysis of ligand–protein contacts generated for compounds **11**–**15** and oxacillin revealed the occurrence of different types of interactions of compound **13** and oxacillin with the allosteric site of PBP2a than those formed by compounds **11**, **12**, **14**, and **15**. Such differences in the interactions between **13** and the remaining compounds were not observed when the molecules tested were bound to the active site of PBP2a, which suggests an allosteric mechanism of cooperative action of **13** with oxacillin. Molecular dynamics simulations supported this hypothesis, since the oxacillin pose in the active site seemed to be unchangeable when compound **13** was bound in the allosteric site.

As stated before, PBP2a has a much lower affinity for most β-lactam antibiotics than the native PBP, and even in the presence of high concentrations of β-lactams, it is capable of a catalyzing transpeptidation reaction that is necessary for the cross-linkage of peptidoglycan chains and bacterial wall biosynthesis [43]. While the native form of PBP which occurs in MSSA strains is inhibited by the β-lactam drugs, PBP2a complements its enzymatic function, simultaneously conferring resistance to this class of anti-infective drugs. The structural studies performed for PBP2a have revealed that this recombinant protein is impervious to inhibition by β-lactam antibiotics, since it exists in a closed conformation form in which an intact β-lactam antibiotic cannot gain access to the active site and trigger its antibacterial effect. This finding has shown that the protein should experience conformational changes at the active site during the catalytic reaction to effectively bind the β-lactam drug and become inactivated [44]. It is well-known that proteins may undergo conformational changes by allosteric modulation that affects binding and the efficacy of the primary ligand [45]. Therefore, it seems that **13**, by interacting with the allosteric domain of PBP2a, induces an opening of its active site, which, in turn, results in oxacillin binding and inhibition of the β-lactam resistance protein [46].

The fact that the compounds tested exclusively influenced the activity of oxacillin, and showed just moderate activity in combination with ampicillin, may be explained by the differences in the susceptibility of these drugs to staphylococcal penicillinases. Ampicillin, due to a high vulnerability to enzymatic hydrolysis, becomes inactivated by specific staphylococcal β-lactamase before binding to conformationally changed PBP2a, and, thus, it seems that compounds were not able to improve its antibacterial effect against penicillinase-positive MRSA strains. On the other hand, the lack of chemosensitizing activity of these compounds in combination with other antibiotics selected for the study may indicate that the molecules did not affect any different mechanisms of resistance than those developed by MRSA against β-lactams. Even though structural analogs of the compounds tested are known as potent modulators of efflux pumps in Gram-positive and Gram-negative bacteria, the lack of a substantial effect, in combination with antibiotics being common substrates for bacterial transporters (i.e., ciprofloxacin and erythromycin), suggests that the interaction of molecules with the efflux system of *S. aureus* is rather unlikely.

Among the compounds of group C, two strong potentiators of oxacillin activity (**18**, **20**) were identified. Docking studies of the binding of chalcogen-containing oxacillin potentiators to the crystal structures of PBP2a indicated that their activity might arise from typical interaction with the active site of the protein. Interestingly, apart from oxacillin adjuvant activity, **18** and **20** were able to enhance the efficacy of erythromycin in MRSA strains resistant to this antibiotic. The fact that compounds elevated the susceptibility of MRSA to erythromycin, which is a well-known substrate of Msr(A) efflux pumps expressed in *S. aureus*, may suggest that the mode of the adjuvant action of these molecules is associated not only with the modulation of PBP2a, but also with the inhibition of a macrolide transporter present in staphylococci. Regrettably, in this study, we were not able to provide evidence to prove this hypothesis.

## 4. Materials and Methods

### 4.1. Crystalographic Studies

Crystals suitable for an X-ray structure analysis for compound **12** were obtained from propan-2-ol, by slow evaporation of the solvent at room temperature.

Data for single crystals of **12** were collected using the Oxford Diffraction SuperNova four circle diffractometer, equipped with the Mo (0.71073 Å) Kα radiation source and graphite monochromator. The phase problem was solved by direct methods using SIR-2014 program [47] and all non-hydrogen atoms were refined anisotropically, using weighted full-matrix least-squares on F^2^. Refinement and further calculations were carried out using SHELXL program [48]. The hydrogen atoms bonded to carbons were included in the structure at idealized positions and were refined using a riding model with U_iso_(H) fixed at 1.2 U_eq_ of C and 1.5 U_eq_ for methyl groups. Hydrogen atoms attached to nitrogen atom were found from the difference Fourier map and refined without any restraints. For molecular graphics, ORTEP [49] and MERCURY [50] programs were used.

**12**: C_30_H_31_N_5_O_4_, M_r_ = 525.60, crystal size = 0.25 × 0.36 × 0.39 mm^3^, monoclinic, space group P2_1_/c, a = 19.1414(4) Å, b = 10.05388(2) Å, c = 14.6239(3), β = 110.538(2)°, V = 2635.4(1) Å^3^, Z = 4, T = 293(2) K, 37086 reflections collected, 6403 unique reflections (R_int_ = 0.0263), R1 = 0.0472, wR2 = 0.1060 [I > 2σ(I)] and R1 = 0.0730, wR2 = 0.1215 [all data].

CCDC 2259874 contain the supplementary crystallographic data. These data can be obtained free of charge from The Cambridge Crystallographic Data Centre via www.ccdc.cam.ac.uk/data_request/cif (accessed on 29 April 2023).

### 4.2. Microbiological Assays

Cation-adjusted Mueller-Hinton (MH II) broth in powder form was provided by Difco Laboratories (Madrid, Spain). The antibiotics oxacillin, ampicillin, erythromycin, ciprofloxacin, and vancomycin were purchased from Sigma-Aldrich (Seelze, Germany). The stock solutions of compounds tested were dissolved in DMSO and stored at −20 °C until assayed. In order to prepare stock solutions, the following solvents were used: deionized MiliQ water to dissolve oxacillin, ampicillin, and vancomycin; 96% (*v*/*v*) ethanol to dissolve erythromycin, as well as deionized MiliQ water, and 18% (*v*/*v*) HCl dropwise to dissolve ciprofloxacin. Antibiotics that were dissolved in deionized MiliQ water were mixed vigorously to homogeneity and filtered through 0.22 µm membranes to ensure the sterility of the solutions.

#### 4.2.1. Bacterial Strains

In vitro antibacterial effectsof fifteen imidazolone derivatives and their anti-MDR properties were elucidated for the panel of two reference methicillin-susceptible (MSSA) and eight methicillin-resistant (MRSA) strains of *S. aureus* with variety of clinical characteristics (Supplementary Materials; Tables S2 and S3).

#### 4.2.2. Susceptibility Testing

In order to quantify direct antibacterial activity of imidazolone derivatives and selected antibiotics ampicillin, erythromycin, ciprofloxacin, and vancomycin against *S. aureus* strains used in the study, MIC (minimum inhibitory concentration) values were determined. This step of the study was necessary for: (i) elucidation whether molecules tested are devoid of antistaphylococcal activity and, thus, cannot become antimicrobial agents by themselves, (ii) determination of the concentrations at which compounds will be tested further for their antibiotic adjuvant potency. MIC values of molecules were assessed via standard microdilution method in cation-adjusted Mueller-Hinton (MH II) broth according to the European Committee on Antimicrobial Susceptibility Testing (EUCAST) guidelines [51]. Experiments were performed in 96-well microtiter plates. MICs were recorded as the lowest concentrations of compounds/antibiotics inhibiting visible growth of bacteria after 18-h incubation at 37 °C. Accurate detection of oxacillin/vancomycin resistance is very challenging due to the frequent coexistence of susceptible and resistant subpopulations within the staphylococcal culture. This phenomenon is termed heteroresistance and contributes to the decreased microbial growth rate of antibiotic-resistant populations in comparison with antibiotic-susceptible ones within bacterial strains. Therefore, the results of susceptibility testing performed with oxacillin or vancomycin in *S. aureus* were read after a full 24 h incubation at 35 °C. Each experiment was conducted in duplicate at least three times.

#### 4.2.3. MIC Reduction Assay

The ability of compounds to enhance the effectiveness of antibiotics was established using microdilution method according to the European Committee on Antimicrobial Susceptibility Testing (EUCAST) guidelines [51]. The compounds were tested at the final concentration not exceeding 1/4 of their respective MICs (usually 1/4 and 1/8 MICs), or at the concentration at which the precipitation of compounds in MH II broth was not observed. Serial dilutions of antibiotics were made in sterile MH II broth, beginning from concentrations which were two-fold higher than the drugs’ MICs. The procedures for the bacterial culture preparation and growth conditions were the same as those described for the susceptibility testing. The potential of compounds to increase the effectiveness of antibiotics was determined by comparing the growth of bacteria in the absence and presence of compounds analyzed. In order to verify if compounds at the concentration tested did not affect bacterial viability, control wells were filled with bacterial culture and a compound studied at the concentration, which was used in combination with an antibiotic. MIC reduction assay for each of compounds was carried out in duplicate in at least three independent experiments. The MIC of an antibiotic in the presence of compound tested represents the value obtained in the majority of repetitions or the mean of all MIC values obtained during the experiments. Antibiotic adjuvant potency of imidazolone derivatives was expressed as an activity gain parameter calculated as a ratio of the MIC of a certain antibiotic to its MIC in conjunction with molecule tested (Equation (1)).

(1) $$A=\left(\frac{MIC_{Ant}}{MIC_{Ant+Comp}}\right)$$

### 4.3. AMES Assay

In the Ames test, compound **13** was dissolved in pure DMSO to obtain the corresponding stock solution (10 mM). Working solutions for compound tested were prepared before the assay in DMSO at 25 μM and 250 μM concentrations. Ampicillin was purchased from Polfa Tarchomin S.A. (Warszawa, Poland). 4-nitroquinoline-*N*-oxide (NQNO), DMSO and bromocresol purple were purchased from Sigma-Aldrich (Seelze, Germany). Doxorubicin hydrochloride was provided by Cayman Chemical (Ann Arbor, Michigan 48108, IN, USA). Beef extract, *L*-histidine monochloride, and *D*-biotin were purchased from Bioshop (Burlington, ON, Canada), whereas peptone from casein was provided by Merck (Darmstadt, Germany). Potassium phosphate monobasic, potassium phosphate, ammonium sulfate, trisodium citrate dehydrate, magnesium sulfate heptahydrate, sodium chloride, and *D*-glucose were purchased from Chempur (Piekary Śląskie, Poland).

The *Salmonella typhimurium* TA 100 strain was used in the Ames test. The strain was selected due to its specificity and sensitivity for a wide range of mutagens. TA100 is characterized by the base-pair substitution (*hisG46* mutation, whose target is GGG). TA 100 strain was purchased from Xenometrix, Switzerland AG (Allschwil, Switzerland).

### 4.4. Cytotoxicity Assay

The cytotoxicity assay was performed according to previously described protocol [52]. The human embryonic kidney HEK-293 (CRL-1573) cell line was obtained from American Type Culture Collection (ATCC). Compound **13** was tested in the concentration range 0.01–100 µM whereas the reference DOXO 0.005–50 µM. The viability of cells was determined after 72 h of incubation with tested compounds by the MTS assay (CellTiter 96^®^ AQueous One Solution Cell Proliferation Assay, Promega, Madison, WI, USA). The absorbance was measured using a microplate reader (Spark, Tecan, Männedorf, Switzerland).

### 4.5. Molecular Modeling

Computational studies were performed for compounds **12**–**21**. Previously, also compounds **7**–**11** were modeled using analogous approach.

For compound **12**, crystal structure was used as an input pose for docking; for the remaining compounds, the three-dimensional conformations and respective protonation states (for pH 7.0 ± 2.0) were generated with the use of LigPrep [53]. At first, all the compounds were docked to the crystal structure of PBP2a protein (PDB code: 3ZFZ [44]). Following the suggested mechanism of interaction of compounds via the allosteric modulation of this target [44,54], dockings were performed in two modes: (i) in which the studied compounds were docked both to the active and allosteric sites (grids were centered at S403 and S240, respectively), and (ii) in which studied compounds were docked to allosteric site (with grid centered at S240) and to the active site (with grid centered at S403); there was oxacillin fitted (the docking was performed in Glide [55,56], and the compounds were docked in extra precision).

The poses with the best Glide docking score were used as starting points for molecular dynamic (MD) simulations. MD simulations were performed in Desmond [53], using TIP3P solvent model [57] and lasted 100 ns; other settings remained default.

The interactions between ligands and PBP2a protein were analyzed using Simulation Interaction Diagram from the Schrodinger Suite.

## 5. Conclusions

In this work, fifteen imidazolone derivatives, **7**–**21**, were evaluated for their ability to potentiate the activity of antibacterial drugs against various MRSA clinical isolates. Results of susceptibility testing indicated that several derivatives tested remarkably increase the effectiveness of oxacillin, and in some cases also ampicillin, against MRSA. Simultaneously, the compounds were devoid of efficacy in reference MSSA strains, which could suggest that the adjuvant action of the compounds arises from interaction with PBP2a. Indeed, by employing docking and molecular dynamic simulations, we have shown that active molecules either allosterically modulate or directly interact with the active site of PBP2a. SAR analysis indicated that the highest ability to restore the antibacterial activity of oxacillin is exerted by the 1-benzhydrylpiperazine derivative of 5-spirofluorenehydantoin (**13**), which reverses oxacillin resistance in most MRSA strains selected for the study. Remarkably, *(Z)*-5-((*E*)-3-phenylallylidene)-2-selenoxo-3-*p*-tolylthiazolidin-4-one (**18**) and *(Z)*-5-benzylidene-3-phenyl-2-selenoxothiazolidin-4-one (**20**) had the capacity to increase the efficacy of not only oxacillin and ampicillin but also erythromycin against resistant strains of MRSA. This finding suggests a dual mode of adjuvant action of **18** and **20** in MRSA, namely, modulation of PBP2a and inhibition of the Msr(A) efflux transporter. Safety studies performed indicated that the most potent oxacillin adjuvant (**13**) has a low mutagenic effect risk, with a much higher risk of cytotoxic actions on eukaryotic cells. The promising antibiotic adjuvant properties of imidazolone derivatives make them good candidates for further development as anti-MDR agents able to restore the activity of antimicrobial drugs against MRSA. Both the antibiotic enhancer action at lower concentration and safety improvements should be the main points for further consideration in extended screening and pharmacomodulations.

## Figures and Tables

**Table 1:** Chemical structures of compounds tested (**7**–**21**).

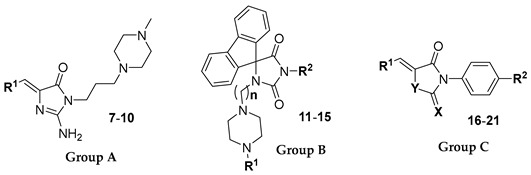						
**Cpd.**	**Group**	**R^1^**	**R^2^**	**n**	**X**	**Y**
**7**	A	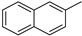	-	-	-	-
**8**	A	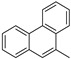	-	-	-	-
**9**	A	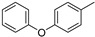	-	-	-	-
**10**	A	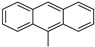	-	-	-	-
**11**	B	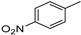	CH_2_COOCH_3_	4	-	-
**12**	B	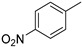	Me	4	-	-
**13**	B	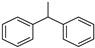	Me	4	-	-
**14**	B	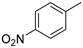	Me	3	-	-
**15**	B	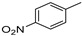	Me	5	-	-
**16**	C	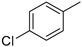	H	-	S	NH
**17**	C	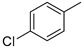	H	-	Se	NH
**18**	C	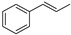	Me	-	Se	S
**19**	C	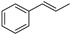	Me	-	S	S
**20**	C	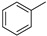	H	-	Se	S
**21**	C	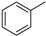	Br	-	S	S

**Table 2:** The parameters of intermolecular interactions of **12**.

D-H···A	H···A (Å)	D···A (Å)	D-H-A (°)	Symmetry Code
C11-H11B···O2	2.35	3.302(2)	168	−x + 1, y + 1/2, −z + 1/2
C21-H21···O4	2.51	3.378(2)	155	−x + 2, −y + 1, −z + 1
C23-H23···O2	2.61	3.523(2)	167	x, −y + 1/2, z − 1/2
C28-H28···O2	2.58	3.462(2)	158	−x + 1, y + 1/2, −z + 1/2

## Data Availability

Most of the data obtained in the study are contained within this article (Main document and Supplementary Materials). Remaining data are available on request from the corresponding author.

## Supplementary Materials

The following supporting information can be downloaded at: https://www.mdpi.com/article/10.3390/antibiotics12111618/s1, Table S1: Chemical characteristics of **7**–**21**; Table S2: Characteristics of S. *aureus* clinical isolates enrolled in the studies; Table S3: Susceptibility of *S. aureus* clinical isolates to antibacterial agents employed in the study; Table S4: Intrinsic antibacterial activities of compounds **7**–**21** against *S. aureus* clinical isolates; Table S5: Effect of compound **7** on MICs of oxacillin and ampicillin against selected *S. aureus* clinical isolates; Table S6: Effect of compound **8** on MICs of oxacillin and ampicillin against selected *S. aureus* clinical isolates; Table S7: Effect of compound **9** on MICs of oxacillin and ampicillin against selected *S. aureus* clinical isolates; Table S8: Effect of compound **10** on MICs of oxacillin and ampicillin against selected *S. aureus* clinical isolates; Table S9: Effect of compound **11** on MICs of oxacillin and ampicillin against selected *S. aureus* clinical isolates; Table S10: Effect of compound **12** on MICs of oxacillin and ampicillin against selected *S. aureus* clinical isolates; Table S11: Effect of compound **13** on MICs of oxacillin and ampicillin against selected *S. aureus* clinical isolates; Table S12: Effect of compound **14** on MICs of oxacillin and ampicillin against selected *S. aureus* clinical isolates; Table S13: Effect of compound **15** on MICs of oxacillin and ampicillin against selected *S. aureus* clinical isolates; Table S14: Effect of compound **16** on MICs of oxacillin and ampicillin against selected *S. aureus* clinical isolates; Table S15: Effect of compound **17** on MICs of oxacillin and ampicillin against selected *S. aureus* clinical isolates; Table S16: Effect of compound **18** on MICs of oxacillin and ampicillin against selected *S. aureus* clinical isolates; Table S17: Effect of compound **19** on MICs of oxacillin and ampicillin against selected *S. aureus* clinical isolates; Table S18: Effect of compound **20** on MICs of oxacillin and ampicillin against selected *S. aureus* clinical isolates; Table S19: Effect of compound **21** on MICs of oxacillin and ampicillin against selected *S. aureus* clinical isolates. Table S20: Effect of compounds **18** and **20** on MICs of ERY against *S. aureus* strains; Table S21: Mutagenic index (MI) and other values for doxorubicin (reference compound) and compound **13**; Figure S1.^1^H NMR for **11;** Figure S2. ^1^H NMR for **21**; Figure S3. ^13^C NMR for **21**.
