# Supplementary material for: Chalcogen-Varied Imidazolone Derivatives as Antibiotic Resistance Breakers in Staphylococcus aureus Strains
Source: Antibiotics (Basel). 2023 Nov 11;12(11):1618. doi: 10.3390/antibiotics12111618 (PMC10669504; doi:10.3390/antibiotics12111618)
Supplement: Supplementary file 1 [file antibiotics-12-01618-s001.zip › antibiotics-2400694-supplementary.pdf]

Imidazolone derivatives as antibiotic resistance breakers in *Staphylococcus aureus* strains

Karolina Witek, Aneta Kaczor, Ewa Żesławska, Sabina Podlewska, Małgorzata Anna Marć, Kinga Czarnota-Łydka, Wojciech Nitek, Gniewomir Latacz, Waldemar Tejchman, Markus Bischoff, Claus Jacob, Jadwiga Handzlik*

Supplementary

Chemical Characteristics of the investigated compounds

*Chemical procedures to obtain compounds **11** and **21** (not published before)*

*Procedure of synthesis of methyl 2-(1′-((4-(4-nitrophenyl)piperazin-1-yl)butyl)-2′,4′-dioxospiro[fluorene-9,5′-imidazolidin]-3′-yl)acetate hydrochloride (**11**)*

Commercially available 4-nitrophenylpiperazine (3 mmol), K_2CO_3 (1.2g) and acetone (10 ml) were stirred for 30 min. Then a solution of the obtained before [23] methyl 2-(1′-(4-bromobutyl)-spiro(fluorene-9,5′ -imidazolidine)-2′,4′-dione-3′-yl)acetate (3 mmol, 1.37g) in acetone (12 ml) was added. The mixture was maintained at reflux for 8 h, left at room temperature for 15 h. The precipitate was filtrated off. The filtrate was concentrated to constant weight. The crude residue was purified by crystallization from EtOH to give a precipitate of the final compound (**11**).

*Procedure of synthesis of (Z)-5-benzylidene-3-(4-bromophenyl)-2-thioxothiazolidin-4-one (**21**)*

3-(4-bromophenyl)-2-((4-bromophenyl)imino)thiazolidin-4-one was boiled with benzaldehyde in a molar ratio 1:1 in the fivefold excess of acetic anhydride for 3-5 h. After cooling the precipitates were filtered off and recrystallized. Then obtained 5-((Z)-benzylidene)-3-(4-bromophenyl)-2-((4-bromophenyl)imino)thiazolidin-4-one was heated with a tenfold excess of dimethyl sulfate under nitrogen for 45-60 min at 120°C. The resulting syrups were dissolved in 25 cm^3 of absolute ethanol and were treated with hydrogen sulfide under nitrogen for 1.5-2 h. Nitrogen was purged for another 1 h to remove the excess of H_2S which was absorbed in the solution of Pb(II) salt. The brightly red precipitates of **21** were filtered off, washed with absolute ethanol and recrystallized.

Table S1. Chemical characteristics of **7**-**21**.

**Compound**	**Name, structure, formula and chemical characteristics** (LC/MS$^+$ or elemental analyses, purity%, ^{1}H-NMR)	**Ref**
**7**	(Z)-2-Amino-3-(3-(4-methylpiperazin-1-yl)propyl)-5-(naphthalen-2-ylmethylene)-3H-imidazol-4(5H)-one hydrochloride 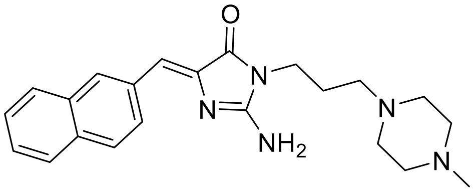	[21]

	$C_{22}H_{28}ClN_5O$ MW 413.94.<br>LC/MS±: purity >99%; t_R = 3.34; (ESI) m/z $[M+H]^+$ 378.28.<br>^{1}H-NMR (DMSO-d6) δ [ppm]: 8.39–8.34 (m, 2H, Ar-5,8-H), 7.85–7.78 (m, 5H, NH_2, Ar-1,3,4-H), 7.47–7.45 (m, 2H, Ar-6,7-H), 6.50 (s, 1H, C = CH), 3.54 (t, J = 6.40Hz, N3-CH_2), 2.45–2.20 (m, 10H, Pip, Pip-CH_2), 2.09 (s, 3H, CH3), 1.68 (qui, J = 6.40Hz, 2H, N-CH_2-C*H_2*)	
**8**	(Z)-2-Amino-3-(3-(4-methylpiperazin-1-yl)propyl)-5-(phenanthren-9-ylmethylene)-3H-imidazol-4(5H)-one hydrochloride<br>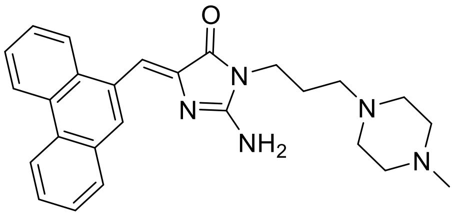<br>$C_{26}H_{30}ClN_5O$ MW 464.00.<br>LC/MS±: purity 99.00% t_R = 4.03, (ESI) m/z $[M+H]^+$ 428.19.<br>^{1}H-NMR (DMSO-d6) δ [ppm]: 8.89–8.86 (m, 2H, Ar-1,10-H), 8.80–8.78 (m, 2H, Ar-4,7-H), 8.24–8.21 (m, 1H, Ar-6-H), 7.89 (s, 2H, NH2), 7.73–7.69 (m, 2H, Ar-2,5-H), 7.68–7.64 (m, 2H, Ar-3,8-H), 6.93 (s, 1H, C = CH), 3.60–3.20 (m, 2H, N3-CH2), 2.45–2.15 (m, 10H, Pip, Pip-CH2), 2.09 (s, 3H, CH3), 1.75 (m, 2H, N-CH2-CH2).	[21]
**9**	(Z)-5-(4-Phenoxybenzylidene)-2-amino-3-(3-(4-methylpiperazin-1-yl)propyl)-3H-imidazol-4(5H)-one<br>hydrochloride 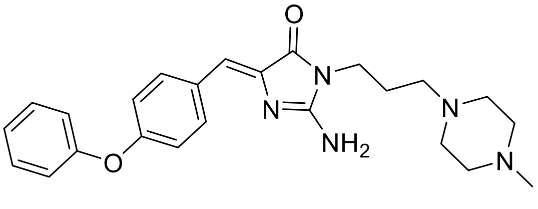<br>$C_{24}H_{30}ClN_5O_2$ MW 455.98.<br>LC/MS±: purity >99% t_R = 4.1, (ESI) m/z $[M+H]^+$ 420.28.<br>^{1}H-NMR (DMSO-d6) δ [ppm]: 7.62–7.57 (m, 3H, NH2, Ph'-4-H), 7.46–7.36 (m, 4H, Ph-2,6-H, Ph'-3,5-H), 7.15–6.95 (m, 4H, Ph-3,5-H, Ph'-2,6-H), 6.72 (s, 1H, C = CH), 3.77 (br. s, 2H, N3-CH2), 3.70–3.14 (m, 10H, Pip, Pip-CH2), 2.79 (s, 3H, CH3), 1.99 (br. s, 2H, N-CH2-CH2).	[21]
**10**	(Z)-2-Amino-5-(anthracen-10-ylmethylene)-3-(3-(4-methylpiperazin-1-yl)propyl)-3H-imidazol-4(5H)-one hydrochloride	[21]

	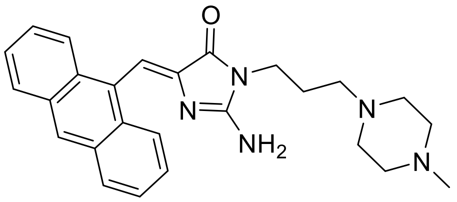<br>$C_{26}H_{30}ClN_5O$ MW 464.00.<br>LC/MS±: purity >99% t_R = 3.89, (ESI) m/z $[M+H]^+$ 428.26.<br>^{1}H-NMR (DMSO-d6) δ [ppm]: 8.61 (br. s, 1H, Ar-5-H), 8.11-8.01 (m, 6H, Ar-1,4,6,9-H, NH2), 7.53–7.52 (m, 4H, Ar-2,3,7,8-H), 7.00 (s, 1H, C = CH), 3.40–3.15 (m, 2H, N3-CH2), 2.56–2.06 (m, 13H, Pip, Pip-CH2, CH3), 1.58–1.52 (m, 2H, N-CH2-CH2).	
**11**	Methyl 2-(1'-((4-(4-nitrophenyl)piperazin-1-yl)butyl)-2',4'-dioxospiro[fluorene-9,5'-imidazolidin]-3'-yl)acetate<br>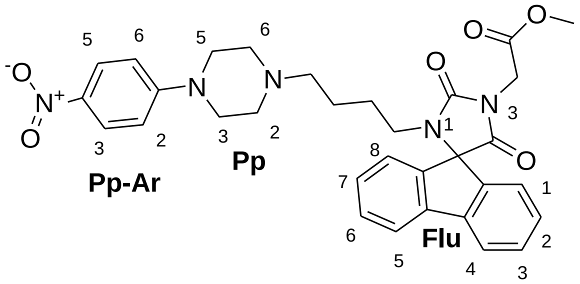<br>hydrochloride<br>$C_{32}H_{33}N_5O_6$ MW 583.63. LC/MS±: purity 95.93%, t_R =5.31 , (ESI) m/z $[M+H]^+$ 584.44<br>R_f = 0.65 (toluene-acetone-MeOH 15:5:1); mp = 193°C<br>^{1}H NMR (DMSO-d6) δ [ppm]: 0.92–1.08 (m, 2H, Pp-CH_2-$C\underline{H}_2$), 1.19–1.25 (t def., 2H, Pp-CH_2), 2.90-2.98 (t def., 4H, Pp-2,6-H), 3.20–3.26 (m, 2H, N1-$CH_2C\underline{H}_2$) 3.40–3.48 (m, 2H, N1-CH_2), 3.73 (s, 3H, OCH_3), 4.19-4.28 (br. s, 2H, N3-CH_2), 4.40–4.46 (m, 4H, Pp-3,5-H), 7.07–7.39 (m, 4H, Pp-Ar-2,6-H, Flu-1,8-H), 7.42–7.55 (m, 4H, Flu-2,3,6,7-H), 7.95–7.97 (m, 2H, Flu-4,5-H), 8.07–8.10 (m, 2H, Pp-Ar-3,5-H).	Compound synthesized based on methods described previously [32]
**12**	3′-Methyl-1′-(4-(4-(4-nitrophenyl)piperazin-1-yl)butyl)spiro[flu-<br>orene-9,5′-imidazolidine]-2′,4′-dione<br>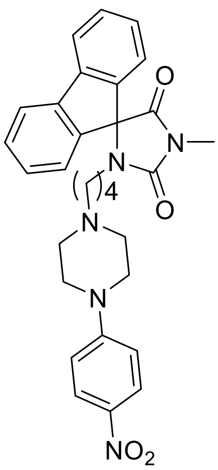	[33]

	$C_{30}H_{31}N_5O_4$ MW 525.24.<br>LC/MS±: purity>95% t_R = 5.01, (ESI) m/z $[M+H]^+$ 526.39.<br>^{1}H NMR (DMSO-d6) δ [ppm]: 0.96–1.01 (q, J = 7.2 Hz, 2H, Pp-CH2- CH2), 1.17–1.22 (q, J = 7.2 Hz, 2H, N1-CH2-CH2), 1.93–1.98 (t, J = 6.9 Hz, 2H, Pp-CH2), 2.23–2.26 (t def, 4H, Pp-2,6-H), 2.88–2.93 (t, J = 7.2 Hz, 2H, N1-CH2), 3.03 (s, 3H, N-CH3), 3.26–3.31 (m, 4H, Pp-3,5-H), 6.96–6.99 (d def, 2H, Pp-Ar-2,6-H), 7.32–7.37 (t def, 2H, Ar-2,7-H), 7.47–7.52 (m, 4H, Ar-1,3,6,8-H), 7.89–7.92 (q def, 2H, Ar-4,5-H), 8.00–8.04 (d def, 2H, Pp-Ar-3,5-H).	
**13**	1′-(4-(4-Benzhydrylpiperazin-1-yl)butyl)-3′-methylspiro[fluorene-9,5′-imidazolidine]-2′,4′-dione hydrochloride<br>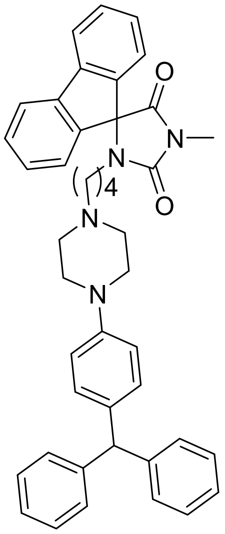<br>$C_{37}H_{39}ClN_4O_2$ MW 607.18.<br>LC/MS±: purity>95% t_R = 6.16, (ESI) m/z $[M+H]^+$ 570.74.<br>^{1}H-NMR (DMSO-d6) δ [ppm]: 0.95 (br.s, 2H, N1-CH2-CH2), 1.47–1.48 (m, 2H, Pp-CH2-CH2), 2.74 (br.s, 2H, Pp-CH2), 2.87 (t, J = 6.8 Hz, 2H, N1-CH2), 3.03 (s, 3H,N3-CH3), 3.39 (br.s, 4H, Pp-2,6-H), 3.67 (br.s, 4H, Pp-3,5-H), 7.28–7.39 (m, 12H, Ar-2,7-H, 2x PpAR-2,3,4,5,6-H), 7.47–7.53 (t def, 4H, Ar-1,3,6,8-H), 7.68 (br.s, 2H, (Ph)2-CH, NH+), 7.90–7.93 (d def, 2H, Ar-4,5-H).	[33]
**14**	3′-Methyl-1′-(3-(4-(4-nitrophenyl)piperazin-1-yl)propyl)spiro [fluorene-9,5′-imidazolidine]-2′,4′-dione	[33]

	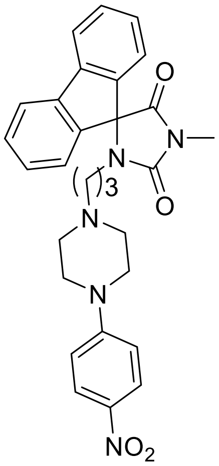<br>$C_{29}H_{29}N_5O_4$ MW 511.57.<br>LC/MS±: purity>95% t_R = 4.92, (ESI) m/z $[M+H]^+$ 512.41.<br>^{1}H NMR (DMSO-d6) δ [ppm]: 1.23–1.28 (qu def, 2H, Pp-CH2-CH2), 2.01–2.08 (qu def, 6H, Pp-CH2, Pp-2,6-H), 2.88–2.93 (t def, 2H, N1-CH2), 3.04 (s, 3H, N3-CH3), 3.19 (br.s, 4H, Pp-3,5-H), 6.93–6.96 (d def, 2H, PpAr-2,6-H),7.33–7.39 (t def, 2H, Ar-2,7-H), 7.49–7.55 (t def, 4H, Ar-1,3,6,8-H), 7.92–7.95 (d def, 2H, Ar-4,5-H), 7.99–8.03 (d def, 2H, PpAr-3,5-H).	
**15**	3′-Methyl-1′-(5-(4-(4-nitrophenyl)piperazin-1-yl)pentyl)spiro [fluorene-9,5′-imidazolidine]-2′,4′-dione<br>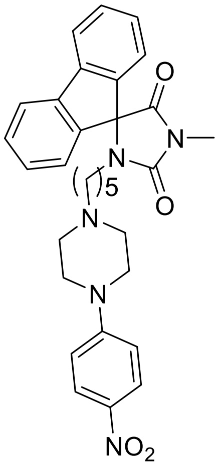<br>$C_{31}H_{33}N_5O_4$ MW 539.62.<br>LC/MS±: purity >95% t_R = 5.21, (ESI) m/z $[M+H]^+$ 540.26.<br>^{1}H NMR (DMSO-d6) δ [ppm]: 0.96–1.08 (m, 6H, N1-CH2-CH2-CH2-CH2), 2.01–2.09 (t def, 2H, Pp-CH2), 2.29–2.33 (t def, 4H, Pp-2,6-H), 2.85–2.89 (t def, 2H, N1-CH2), 3.03 (s, 3H,N3-CH3), 3.28–3.37 (br.s, 4H, Pp-3,5-H), 6.95–7.00 (d def, 2H, PpAr-2,6-H), 7.32–7.37 (t def, 2H, Ar-2,7-H), 7.46–7.53 (m, 4H, Ar-1,3,6,8-H), 7.89–7.93 (d def, 2H, Ar-4,5-H), 8.00–8.05 (d def, 2H, PpAr-3,5-H).	[33]
**16**	(Z)-5-(4-chlorobenzylidene)-3-phenyl-2-thioxoimidazolidin-4-one	[35]

	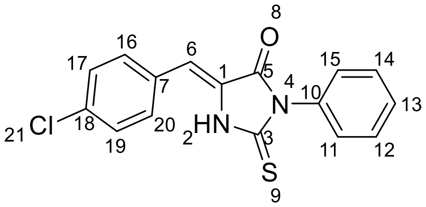<br>$C_{16}H_{11}ClN_2OS$, MW 314.79.<br>LC/MS±: purity > 99%; tR = 9.22; (ESI) m/z $[M+H]^+$ 315.259.<br>^{1}H NMR (DMSO-d6) δ [ppm]: 12.66 (s, 1H, NH), 7.84 (d, 2H, J = 8.7 Hz, Ar-16,20-H), 7.54 – 7.43 (m, 5H, Ar-11,12,13,14,15-H), 7.39 (dd, 2H, J = 8.2, 1.1 Hz, Ar-17,19-H), 6.68 (s, 1H, C = CH).	
**17**	(Z)-5-(4-chlorobenzylidene)-3-phenyl-2-selenoxoimidazolidin-4-one<br>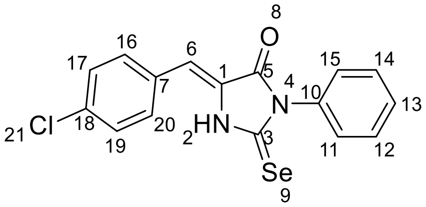<br>$C_{16}H_{11}ClN_2OSe$, MW 361.70.<br>LC/MS±: purity >99%; tR = 9.22; (ESI) m/z $[M+H]^+$ 362.887.<br>^{1}H NMR (DMSO-d6) δ [ppm]: 13.18 (s, 1H, NH), 7.90 (d, 2H, J = 8.5 Hz, Ar- 16,20-H), 7.57 – 7.50 (m,5H, Ar- 11,12,13,14,15-H), 7.41 – 7.38 (m, 2H, Ar-17,19-H).	[35]
**18**	(Z)-5-((E)-3-phenylallylidene)-2-selenoxo-3-(p-<br>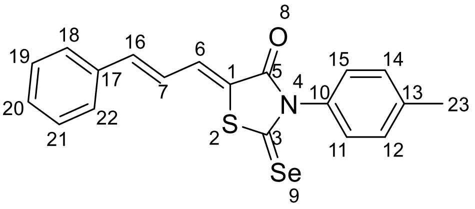<br>tolyl)thiazolidin-4-one<br>$C_{19}H_{15}NOSSe$, MW 384.37.<br>LC/MS±: purity 95%; tR = 9.99; (ESI) m/z $[M+H]^+$ 385.836<br>^{1}H NMR (DMSO-d6) δ [ppm]: δ 7.74 (m, 2H, Ar-18,22-H), 7.64 (d, 1H,J = 11.6 Hz, C = CH16), 7.49 (d, 1H, J = 15.1 Hz, C=CH6), 7.45 – 7.41 (m, 3H, Ar-19,20,21-H), 7.35 (d, 2H, J = 8.0 Hz, Ar-11,15-H), 7.27 (d, 2H, J = 8.2 Hz, , Ar-12,14-H), 7.19 (dd, 1H, J = 15.1, 11.6 Hz, C=CH7), 2.37 (s, 3H, $-CH_3$).	[36]
**19**	(Z)-5-((E)-3-phenylallylidene)-2-thioxo-3-(p-tolyl)thiazolidin-4-one<br>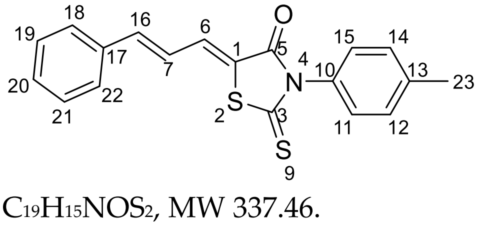<br>$C_{19}H_{15}NOS_2$, MW 337.46.<br>LC/MS±: purity >99%; tR = 3.34; (ESI) m/z $[M+H]^+$ 338.275.	[36]

	^{1}H NMR (DMSO-d6) δ [ppm]: δ 7.73 (d, 2H, J = 6.5 Hz, Ar-18,22-H), 7.56 (d, 1H, J = 11.5 Hz, C = CH16), 7.46 – 7.40 (m, 4H, Ar-19,20,21-H, C=CH6), 7.34 (d, 2H ,J = 8.3 Hz, Ar-11,15-H), 7.25 (d, 2H, J = 8.3 Hz, Ar-12,14-H), 7.17 (dd, 1H, J = 15.2, 11.6 Hz, C=CH7), 2.37 (s, 3H, $-CH_3$).	
**20**	(Z)-5-benzylidene-3-phenyl-2-selenoxothiazolidin-4-one<br>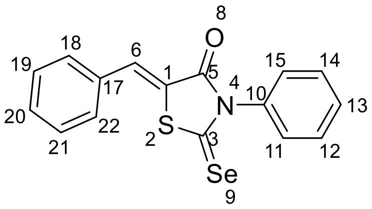<br>$C_{16}H_{11}NOSSe$, MW 344.30.<br>Elemental analysis %(calculated/found):<br>C 55.8/56.4, H 3.2/3.1, N 4.1/3.9. Purity >95%<br>^{1}H NMR in $CDCl_3$ δ [ppm]: δ 7.13-7.73 (m, 10H, Ar), 7.90 (s, 1H, C=CH)	[36]
**21**	(Z)-5-benzylidene-3-(4-bromophenyl)-2-thioxothiazolidin-4-one<br>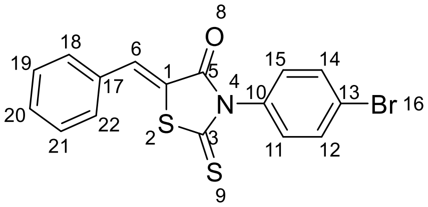<br>$C_{16}H_{10}BrNOS_2$, MW 376.29.<br>LC/MS±: purity > 99%; tR = 9.95; (ESI) m/z $[M+H]^+$ 377.987. HRMS purity > 99%; tR = 9.41 [ESI+]: $[M+H]^+$ found: 377.854.^{1}H NMR (DMSO-d6) δ [ppm]: δ 7.86 (s, 1H, C = CH), 7.78 (d, 2H, J = 8.8 Hz, Ar-11,15-H), 7.69 (d, 2H, J = 7.4 Hz, Ar-18,22-H), 7.61 – 7.52 (m, 3H, Ar-19,20,21-H), 7.42 (d, 2H, J = 8.8 Hz, Ar-12,14-H). ^{13}C NMR (DMSO-d6) δ [ppm]: NMR (126 MHz,) δ 194.44, 167.30, 135.03, 133.55, 133.31, 132.95, 131.60, 131.54, 131.16, 130.14, 123.83, 123.42.	Compound synthesized using methods described previously [36]

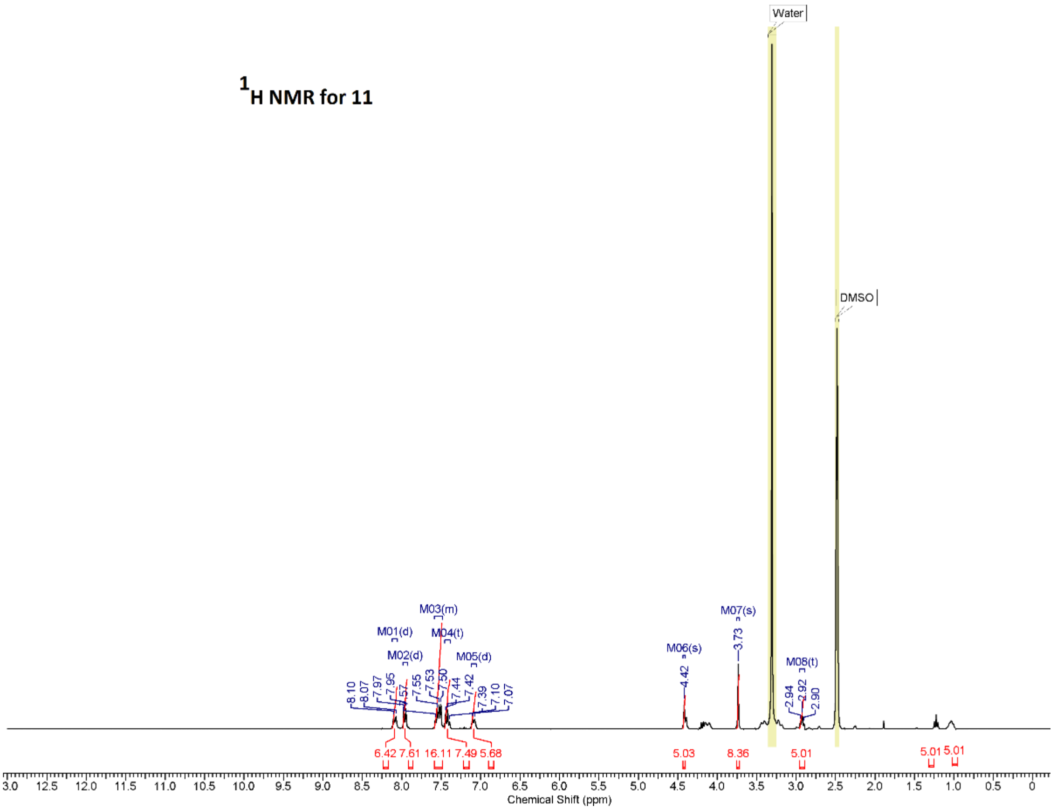

Figure S1. ^{1}H NMR for **11**.

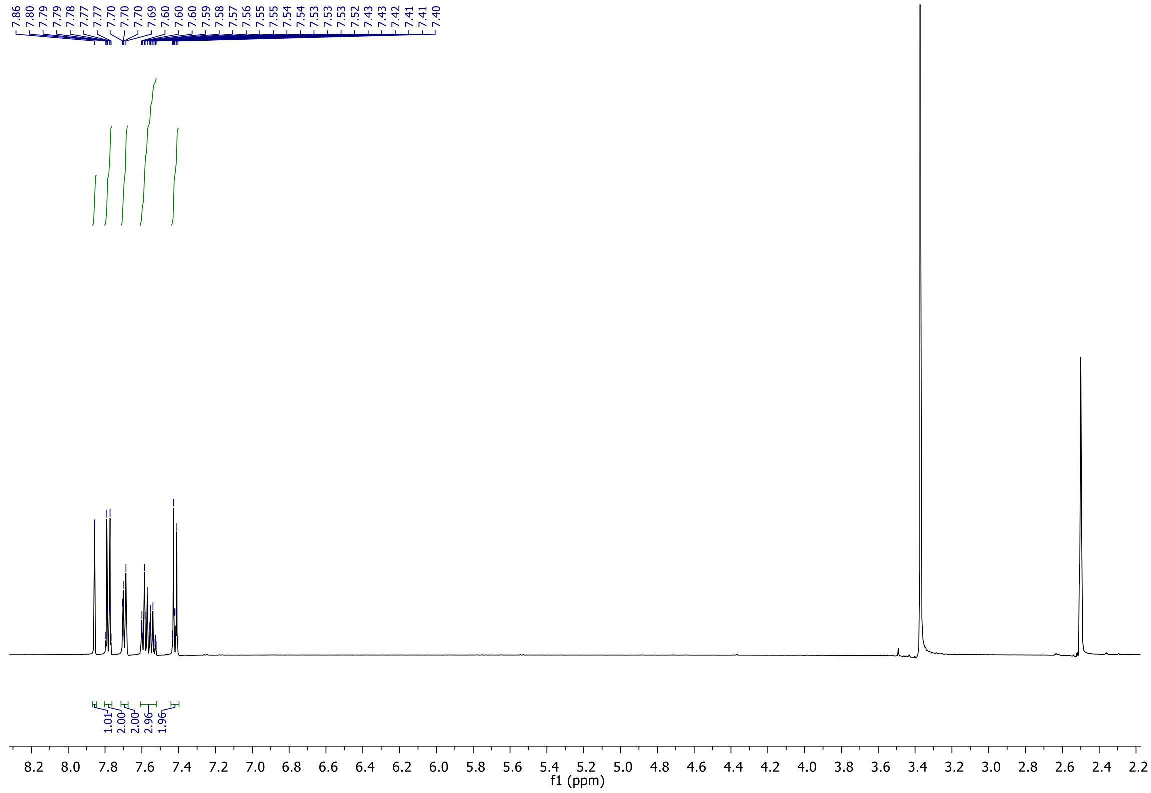

Figure S2. ^{1}H-NMR for **21**.

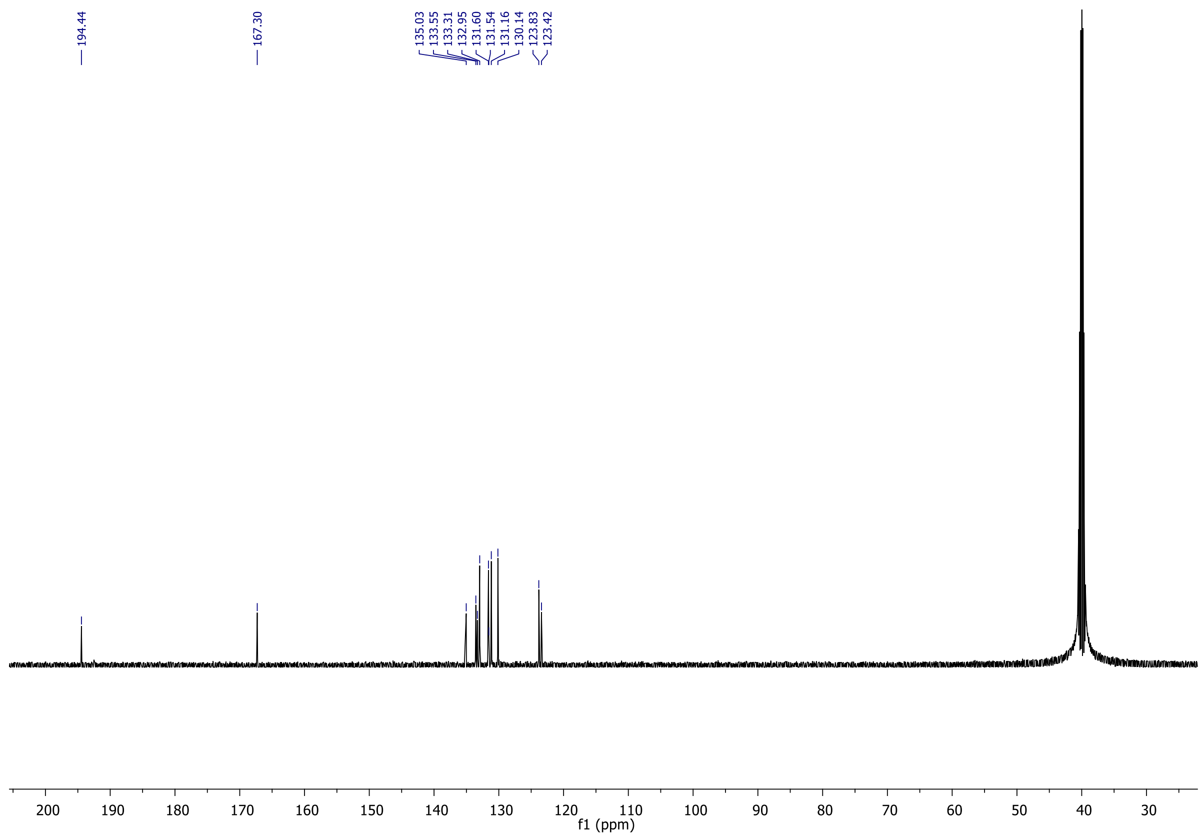

Figure S3. ^{13}C NMR for **21**.

[21] Kaczor, A.; Witek, K.; Podlewska, S.; Czekajewska, J.; Lubelska, A.; Żesławska, E.; Nitek, W.; Latacz, G.; Alibert, S.; Pagès, J.M.; et al. 5-Arylideneimidazolones with Amine at Position 3 as Potential Antibiotic Adjuvants against Multidrug Resistant Bacteria. *Molecules* **2019**, *24*, doi:10.3390/molecules24030438

[32] Żesławska, E.; Kincses, A.; Spengler, G.; Nitek, W.; Wyrzuc, K.; Kieć-Kononowicz, K.; Handzlik, J. The 5-Aromatic Hydantoin-3-Acetate Derivatives as Inhibitors of the Tumour Multidrug Resistance Efflux Pump P-Glycoprotein (ABCB1): Synthesis, Crystallographic and Biological Studies. *Bioorg Med Chem* **2016**, *24*, 2815–2822, doi:10.1016/j.bmc.2016.04.055.

[33] Żesławska, E.; Kucwaj-Brysz, K.; Kincses, A.; Spengler, G.; Szymańska, E.; Czopek, A.; Marć, M.A.; Kaczor, A.; Nitek, W.; Domínguez-Álvarez, E.; et al. An Insight into the Structure of 5-Spiro Aromatic Derivatives of Imidazolidine-2,4-Dione, a New Group of Very Potent Inhibitors of Tumor Multidrug Resistance in T-Lymphoma Cells. *Bioorg Chem* **2021**, *109*, doi:10.1016/j.bioorg.2021.104735.

[35] Korohoda, M. Introduction of Selenium into Heterocycliccompounds. 1. Synthesis of 3-Aryl-2-Selenohydantoins with Double-Bond at C-5. Pol J Chem **1980**, 54, 683–692.

[36] Tejchman, W.; Korohoda, M.J. Introduction of Selenium to Heterocyclic Compounds. Part VI. Synthesis of 3-Aryl-5-Benzylidene- and 3-Aryl-5-Cinnamylidene-2-Selenorhodanines. *Polish J. Chem.* **1996**, *70*, 1124–1134.

Microbiological assays

Table S2. Characteristics of S. *aureus* clinical isolates enrolled in the studies.

***Staphylococcus aureus* strain**	**Relevant phenotype***
MM-O058	Clinical isolate, CC121, MSSA, MDR
MM-N072	Clinical isolate, CC152, MSSA, MDR
LG-N017	Clinical isolate, CC5, MRSA, MDR
MM-O021	Clinical isolate, CC8, MRSA, MDR
R45-CC45	Clinical isolate, CC45, MRSA
R46-CC22	Clinical isolate, CC22, MRSA
USA300 LAC	Clinical isolate, CC8, CA-MRSA, MDR
5328	Clinical isolate, CC398, LA-MRSA
COL	Clinical isolate, MRSA
Mu50	Clinical isolate, CC5, MRSA, VISA

*CC, clonal complex; MSSA, methicillin-susceptible *S. aureus*; MRSA, methicillin-resistant *S. aureus*; CA-MRSA, community-acquired MRSA; LA-MRSA, livestock-associated MRSA; VISA, vancomycin-intermediate *S. aureus;* MDR, multidrug-resistant.

Table S3. Susceptibility of *S. aureus* clinical isolates to antibacterial agents employed in the studies.

Strain	**MIC of antibiotics [μg/ml]**				
	**OXA**	**AMP**	**ERY**	**CIP**	**VAN**
MM-O058	0.25 (0.0006)	8 (0.0229)	> 256 (0.3488)	0.25 (0.0006)	ND
MM-N072	0.25 (0.0006)	32 (0.0916)	≥ 256 (0.3488)	0.25 (0.0006)	ND
LG-N017	12 (0.0283)	16 (0.0458)	8 (0.0109)	0.5 (0.0013)	ND
MM-O021	48 (0.1134)	128 (0.3663)	> 256 (0.3488)	0.25 (0.0006)	ND
R45-CC45	4 (0.0094)	48 (0.1374)	1 (0.0014)	0.25 (0.0006)	ND
R46-CC22	64 (0.1512)	32 (0.0916)	0.5 (0.0007)	64 (0.1659)	ND
USA300 LAC	12 (0.0283)	64 (0.1832)	64 (0.0872)	8 (0.0207)	ND
5328	4 (0.0094)	64 (0.1832)	> 256 (0.3488)	0.25 (0.0006)	ND
COL	128 (0.3023)	8 (0.0229)	0.25 (0.0003)	0.25 (0.0006)	ND
Mu50	256 (0.6046)	32 (0.0916)	> 256 (0.3488)	32 (0.0829)	8 (0.0055)

OXA, oxacillin; AMP, ampicillin; ERY, erythromycin; CIP, ciprofloxacin; VAN, vancomycin. Values in brackets represent MIC values in mM. ND – not determined.

Table S4. Intrinsic antibacterial activities of compounds **7-21** against *S. aureus* clinical isolates.

**Compound**	**MIC of compound [mM]***									
	MM-O058	MM-N072	LG-N017	MM-O021	R45-CC45	R46-CC22	USA300 LAC	5328	COL	MU50
**7**	> 1	> 1	> 1	> 1	> 1	> 1	> 1	> 1	> 1	> 1
**8**	0.25	0.25	0.25-0.5	0.25-0.5	0.25	0.25	0.25	0.25	0.5	0.25-0.5
**9**	0.5	0.5-1	1	> 1	1	1	1	1	> 0.5	0.5
**10**	0.5	0.5	1	1	0.5	0.5	0.25	0.5	1	0.5
**11**	≥ 0.5	≥ 0.5	≥ 0.5	≥ 0.5	≥ 0.5	≥ 0.5	≥ 0.5	≥ 0.5	≥ 0.5	≥ 0.5
**12**	0.25-0.5	0.25	0.5	0.5	0,5	1	1	0.5	0.5	0.5
**13**	0.25	0.25	0.25	0.25	0.25	0.25	0.25	0.25	0.25	0.25
**14**	0.25	0.25	0.25	0.25	0.25	0.25	0.25	0.25	0.25	0.25
**15**	0.5	0.5	0.5	0.5	0.5	0.5	0.5	0.5	0.5	0.25
**16**	> 0.25	> 0.25	> 0.25	> 0.25	0.25	> 0.25	> 0.25	> 0.25	> 0.25	0.25
**17**	> 0.25	> 0.25	> 0.25	> 0.25	> 0.25	> 0.25	> 0.25	> 0.25	> 0.25	> 0.25
**18**	> 0.125	> 0.125	> 0.125	> 0.125	> 0.125	>0.125	> 0.125	> 0.125	>0.125	>0.125
**19**	> 0.25	> 0.25	> 0.25	> 0.25	> 0.25	> 0.25	> 0.25	> 0.25	> 0.25	> 0.25
**20**	> 0.125	> 0.125	> 0.125	> 0.125	0.0625	>0.125	> 0.125	> 0.125	>0.125	>0.125
**21**	0.25	0.25	0.25	0.25	> 0.25	> 0.25	> 0.25	> 0.25	0.25	> 0.25

*Assessment of the exact MICs of the compounds 7, 11, 16-23 was not always possible due to their precipitation in bacterial suspension.

Table S5. Effect of compound **7** MICs of oxacillin (OXA) and ampicillin (AMP) against selected *S. aureus* clinical isolates.

		**OXA**		**AMP**	
**Strain**	**Concentration of compound [mM]**	**Numerical value of reduction [μg/ml]**	**Activity gain [A]**	**Numerical value of reduction [μg/ml]**	**Activity gain [A]**
**MM-O058**	0.25	-	no effect	-	no effect
	0.5	-	no effect	from 8 to 4	2
**MM-N072**	0.25	-	no effect	from 32 to 16	2
	0.5	from 0.25 to 0.125	2	from 32 to 16	2
**LG-N017***	0.25	**from 12 to 2**	6	from 16 to 4	4
	0.5	**from 12 to 0.25**	48	from 16 to 1.5	10.7
**MM-O021***	0.25	from 48 to 8	6	from 128 to 64	2
	0.5	**from 48 to 1.5**	32	from 128 to 32	4
**R45-CC45***	0.25	**from 4 to 1**	4	from 48 to 8	6
	0.5	**from 4 to 0.25**	16	from 48 to 2	24
**R46-CC22***	0.25	from 64 to 32	2	from 32 to 16	2
	0.5	from 64 to 8	8	from 32 to 8	4
**USA300 LAC***	0.25	**from 12 to 1.5**	8	-	no effect
	0.5	**from 12 to 0.5**	24	from 64 to 16	4
**5328***	0.25	**from 4 to 1**	4	from 64 to 32	2
	0.5	**from 4 to 0.25**	16	from 64 to 16	4
**COL***	0.25	from 128 to 32	4	-	no effect
	0.5	**from 128 to 2**	64	from 8 to 2	4
**Mu50***	0.25	from 256 to 32	8	-	no effect
	0.5	from 256 to 4	64	from 32 to 4	8

*MRSA strain; values in bold correspond to a restoration of OXA activity against MRSA.

Table S6. Effect of compound **8** on MICs of OXA and AMP against selected *S. aureus* clinical isolates.

		**OXA**		**AMP**	
**Strain**	**Concentration of compound [mM]**	**Numerical value of reduction [μg/ml]**	**Activity gain [A]**	**Numerical value of reduction [μg/ml]**	**Activity gain [A]**
**MM-O058**	0.0625	-	no effect	from 8 to 6	1.3
**MM-N072**	0.0625	from 0.25 to 0.125	2	from 32 to 16	2
**LG-N017***	0.0625	from 12 to 4	3	-	no effect
	0.125	**from 12 to 2**	6	from 16 to 12	1.3
**MM-O021***	0.0625	from 48 to 16	3	-	no effect
	0.125	**from 48 to 0.25**	192	from 128 to 8	16
**R45-CC45***	0.0625	**from 4 to 1.5**	2.7	from 48 to 16	3
**R46-CC22***	0.0625	from 64 to 32	2	from 32 to 24	1.3
**USA300 LAC***	0.0625	**from 12 to 1.5**	8	-	no effect
**5328***	0.0625	**from 4 to 0.75**	5.3	from 64 to 32	2
**COL***	0.0625	from 128 to 32	4	-	no effect
	0.125	**from 128 to 2**	64	from 8 to 4	2
**Mu50***	0.0625	from 256 to 64	4	-	no effect
	0.125	from 256 to 32	8	from 32 to 16	-

*MRSA strain; values in bold correspond to a restoration of OXA activity against MRSA.

Table S7. Effect of compound **9** on MICs of OXA and AMP against selected *S. aureus* clinical isolates.

		**OXA**		**AMP**	
**Strain**	**Concentration of compound [mM]**	**Numerical value of reduction [µg/ml]**	**Activity gain [A]**	**Numerical value of reduction [µg/ml]**	**Activity gain [A]**
**MM-O058**	0.125	-	no effect	-	no effect
**MM-N072**	0.25	-	no effect	from 32 to 16	2
**LG-N017***	0.125	**from 12 to 2**	6	-	no effect
	0.25	**from 12 to 0.25**	24	from 16 to 8	2
**MM-O021***	0.25	**from 48 to 2**	24	from 128 to 49	2.5
	0.5	**from 48 to 0.25**	192	from 128 to 7	18
**R45-CC45***	0.125	**from 4 to 1**	4	from 48 to 16	3
	0.25	**from 4 to 0.25**	16	from 48 to 4	12
**R46-CC22***	0.125	from 64 to 16	4	-	no effect
	0.25	from 64 to 4	16	from 32 to 16	2
**USA300 LAC***	0.125	**from 12 to 1.5**	8	-	no effect
	0.25	**from 12 to 0.25**	48	-	no effect
**5328***	0.125	**from 4 to 2**	2	from 64 to 48	1.3
	0.25	**from 4 to 0.5**	8	from 64 to 32	2
**COL***	0.125	from 128 to 32	4	-	no effect
	0.25	**from 128 to 2**	64	from 8 to 2	4
	0.0625	from 256 to 32	8	-	no effect
**Mu50***	0.125	from 256 to 8	32	from 32 to 16	2

*MRSA strain; values in bold correspond to a restoration of OXA activity against MRSA.

Table S8. Effect of compound **10** on MICs of OXA and AMP against selected *S. aureus* clinical isolates.

		**OXA**		**AMP**	
**Strain**	**Concentration of compound [mM]**	**Numerical value of reduction [µg/ml]**	**Activity gain [A]**	**Numerical value of reduction [µg/ml]**	**Activity gain [A]**
**MM-O058**	0.125	-	no effect	-	no effect
**MM-N072**	0.125	-	no effect	-	no effect
**LG-N017***	0.125	from 12 to 4	3	from 16 to 4	4
	0.25	**from 12 to 1**	12	from 16 to 4	4
**MM-O021***	0.25	from 48 to 8	8	from 128 to 32	4
**R45-CC45***	0.125	**from 4 to 2**	2	from 48 to 16	3
**R46-CC22***	0.125	from 64 to 16	4	from 32 to 16	2
**USA300 LAC***	0.0625	**-**	8	-	no effect
**5328***	0.125	**-**	no effect	-	no effect
**COL***	0.125	-	no effect	-	no effect
	0.25	**-**	no effect	-	-
**Mu50***	0.125	from 256 to 16	16	from 32 to 16	2

*MRSA strain; values in bold correspond to a restoration of OXA activity against MRSA.

Table S9. Effect of compound **11** on MICs of OXA and AMP against selected *S. aureus* clinical isolates.

		OXA		AMP	
**Strain**	**Concentration of compound [mM]**	**Numerical value of reduction [µg/ml]**	**Activity gain [A]**	**Numerical value of reduction [µg/ml]**	**Activity gain [A]**
**MM-O058**	0.0625	-	no effect	-	no effect
	0.125	-	no effect	-	no effect
**MM-N072**	0.0625	-	no effect	-	no effect
	0.125	-	no effect	from 32 to 16	2
**LG-N017***	0.0625	from 12 to 4	3	-	no effect
	0.125	from 12 to 4	3	from 16 to 8	2
**MM-O021***	0.0625	from 48 to 6	8	-	no effect
	0.125	from 48 to 4	12	-	no effect
**R45-CC45***	0.0625	**from 4 to 2**	2	from 48 to 32	1.5
	0.125	**from 4 to 0.5**	8	from 48 to 16	3
**R46-CC22***	0.0625	-	no effect	from 32 to 16	2
	0.125	from 64 to 32	2	from 32 to 8	4
**USA300 LAC***	0.0625	from 12 to 6	2	-	no effect
	0.125	**from 12 to 1**	12	from 64 to 24	2.7
**5328***	0.0625	**-**	no effect	-	no effect
	0.125	-	no effect	-	no effect
**COL***	0.0625	-	no effect	-	no effect
	0.125	-	no effect	-	no effect
**Mu50***	0.0625	from 256 to 64	4	-	no effect
	0.125	from 256 to 64	4	-	no effect

*MRSA strain; values in bold correspond to a restoration of OXA activity against MRSA.

Table S10. Effect of compound **12** on MICs of OXA and AMP against selected *S. aureus* clinical isolates.

		OXA		AMP	
**Strain**	**Concentration of compound [mM]**	**Numerical value of reduction [µg/ml]**	**Activity gain [A]**	**Numerical value of reduction [µg/ml]**	**Activity gain [A]**
**MM-O058**	0.0625	-	no effect	-	no effect
	0.125	-	no effect	-	no effect
**MM-N072**	0.0625	-	no effect	-	no effect
**LG-N017***	0.0625	-	no effect	from 16 to 12	1.3
	0.125	**from 12 to 0.75**	16	from 16 to 12	1.3
**MM-O021***	0.0625	from 48 to 24	2	-	no effect
	0.125	**from 48 to 2**	24	-	no effect
**R45-CC45***	0.125	**from 4 to 1**	4	from 48 to 32	1.5
	0.25	**from 4 to 0.5**	8	from 48 to 16	3
**R46-CC22***	0.125	-	no effect	from 32 to 16	2
	0.25	from 64 to 32	2	from 32 to 8	4
**USA300 LAC***	0.125	**from 12 to 0.25**	48	from 64 to 48	1.3
**5328***	0.125	-	no effect	-	no effect
	0.25	-	no effect	from 64 to 12	5.3
**COL***	0.0625	-	no effect	-	no effect
	0.125	-	no effect	-	no effect

**Mu50***	0.0625	from 256 to 32	8	-	no effect
	0.125	from 256 to 32	8	-	no effect

*MRSA strain; values in bold correspond to a restoration of OXA activity against MRSA.

Table S11. Effect of compound **13** on MICs of OXA and AMP against *S. aureus* clinical isolates.

		**OXA**		**AMP**	
**Strain**	**Concentration of compound [mM]**	**Numerical value of reduction [µg/ml]**	**Activity gain [A]**	**Numerical value of reduction [µg/ml]**	**Activity gain [A]**
**MM-O058**	0.0625	from 0.25 to 0.125	2	from 8 to 4	2
**MM-N072**	0.0625	from 0.25 to 0.125	2	from 32 to 16	2
**LG-N017***	0.0625	**from 12 to 0.25**	48	from 16 to 4	4
**MM-O021***	0.0625	**from 48 to 1.5**	32	from 128 to 64	2
**R45-CC45***	0.0625	**from 4 to 0.125**	32	from 48 to 8	6
**R46-CC22***	0.0625	**from 64 to 2**	32	from 32 to 8	4
**USA300 LAC***	0.0625	**from 12 to 0.25**	48	from 64 to 32	2
**5328***	0.0625	**from 4 to 0.125**	32	from 64 to 32	2
**COL***	0.0625	from 128 to 64	2	-	no effect
**Mu50***	0.0625	from 256 to 64	2	from 32 to 16	2

*MRSA strain; values in bold correspond to a restoration of OXA activity against MRSA.

Table S12. Effect of compound **14** on MICs of OXA and AMP against *S. aureus* clinical isolates.

		**OXA**		**AMP**	
**Strain**	**Concentration of compound [mM]**	**Numerical value of reduction [µg/ml]**	**Activity gain [A]**	**Numerical value of reduction [µg/ml]**	**Activity gain [A]**
**MM-O058**	0.0625	-	2	from 8 to 4	2
**MM-N072**	0.0625	-	2	from 32 to 16	2
**LG-N017***	0.0625	**from 12 to 2**	6	-	no effect
**MM-O021***	0.0625	from 48 to 12	4	-	no effect
**R45-CC45***	0.0625	**from 4 to 1**	4	-	no effect
**R46-CC22***	0.0625	-	no effect	from 32 to 24	1.5
**USA300 LAC***	0.0625	**from 12 to 2**	6	-	no effect
**5328***	0.0625	**from 4 to 1.5**	2.7	-	no effect
**COL***	0.0625	-	no effect	-	no effect
**Mu50***	0.0625	from 256 to 128	2	-	no effect

*MRSA strain; values in bold correspond to a restoration of OXA activity against MRSA.

Table S13. Effect of compound **15** on MICs of OXA and AMP against *S. aureus* clinical isolates.

		**OXA**		**AMP**	
**Strain**	**Concentration of compound [mM]**	**Numerical value of reduction [µg/ml]**	**Activity gain [A]**	**Numerical value of reduction [µg/ml]**	**Activity gain [A]**
**MM-O058**	0.0625	-	no effect	-	no effect
**MM-N072**	0.0625	-	no effect	-	no effect
**LG-N017***	0.0625	-	no effect	-	no effect
**MM-O021***	0.0625	-	no effect	-	no effect
**R45-CC45***	0.0625	-	-	-	no effect
**R46-CC22***	0.0625	-	no effect	-	no effect
**USA300 LAC***	0.0625	from 12 to 8	1.5	-	no effect

**5328***	0.0625	-	no effect	-	no effect
**COL***	0.0625	-	no effect	-	no effect
**Mu50***	0.0625	-	no effect	-	no effect

*MRSA strain

Table S14. Effect of compound **16** on MICs of OXA and AMP against *S. aureus* strains.

		**OXA**		**AMP**	
**Strain**	**Concentration of compound [mM]**	**Numerical value of reduction [μg/ml]**	**Activity gain [A]**	**Numerical value of reduction [μg/ml]**	**Activity gain [A]**
**MM-O058**	0.03125	-	no effect	-	no effect
	0.0625	-	no effect	-	no effect
**MM-N072**	0.03125	-	no effect	-	no effect
	0.0625	-	no effect	-	no effect
**LG-N017***	0.03125	-	no effect	-	no effect
	0.0625	-	no effect	-	no effect
**MM-O021***	0.03125	-	no effect	-	no effect
	0.0625	-	no effect	-	no effect
**R45-CC45***	0.03125	-	no effect	-	no effect
	0.0625	-	no effect	-	no effect
**R46-CC22***	0.03125	-	no effect	-	no effect
	0.0625	-	no effect	-	no effect
**USA300 LAC***	0.03125	-	no effect	-	no effect
	0.0625	-	no effect	-	no effect
**5328***	0.03125	-	no effect	-	no effect
	0.0625	-	no effect	-	no effect
**COL***	0.03125	-	no effect	-	no effect
	0.0625	-	no effect	-	no effect
**Mu50***	0.03125	-	no effect	-	no effect
	0.0625	-	no effect	-	no effect

*MRSA strain

Table S15. Effect of compound **17** on MICs of OXA and AMP against *S. aureus* strains.

		**OXA**		**AMP**	
**Strain**	**Concentration of compound [mM]**	**Numerical value of reduction [μg/ml]**	**Activity gain [A]**	**Numerical value of reduction [μg/ml]**	**Activity gain [A]**
**MM-O058**	0.03125	-	no effect	-	no effect
	0.0625	-	no effect	-	no effect
**MM-N072**	0.03125	-	no effect	-	no effect
	0.0625	-	no effect	-	no effect
**LG-N017***	0.03125	-	no effect	-	no effect
	0.0625	-	no effect	-	no effect
**MM-O021***	0.03125	-	no effect	-	no effect
	0.0625	-	no effect	-	no effect
**R45-CC45***	0.03125	-	no effect	-	no effect
	0.0625	-	no effect	-	no effect
**R46-CC22***	0.03125	-	no effect	-	no effect
	0.0625	-	no effect	-	no effect
**USA300 LAC***	0.03125	-	no effect	-	no effect
	0.0625	-	no effect	-	no effect
**5328***	0.03125	-	no effect	-	no effect

	0.0625	-	no effect	-	no effect
**COL***	0.03125	-	no effect	-	no effect
	0.0625	-	no effect	-	no effect
**Mu50***	0.03125	-	no effect	-	no effect
	0.0625	-	no effect	-	no effect

*MRSA strain

Table S16. Effect of compound **18** on MICs of OXA and AMP against *S. aureus* strains.

		OXA		AMP	
**Strain**	**Concentration of compound [mM]**	**Numerical value of reduction [µg/ml]**	**Activity gain [A]**	**Numerical value of reduction [µg/ml]**	**Activity gain [A]**
**MM-O058**	0.03125	-	no effect	from 8 to 4	2
	0.0625	-	no effect	from 8 to 4	2
**MM-N072**	0.03125	-	no effect	from 32 to 16	2
	0.0625	-	no effect	from 32 to 16	2
**LG-N017***	0.03125	**from 12 to 0.5**	24	from 16 to 8	2
	0.0625	**from 12 to 0.25**	48	from 16 to 4	4
**MM-O021***	0.03125	from 48 to 16	3	from 128 to 64	2
	0.0625	from 48 to 4	12	from 128 to 64	2
**R45-CC45***	0.03125	**from 4 to 1**	4	from 48 to 16	3
	0.0625	**from 4 to 0.25**	16	from 48 to 8	6
**R46-CC22***	0.03125	**from 64 to 2**	32	-	no effect
	0.0625	**from 64 to 1**	64	-	no effect
**USA300 LAC***	0.03125	**from 12 to 0.5**	24	-	no effect
	0.0625	**from 12 to 0.25**	48	from 64 to 16	4
**5328***	0.03125	-	no effect	from 64 to 32	2
	0.0625	-	no effect	from 64 to 16	4
**COL***	0.03125	-	no effect	-	no effect
	0.0625	-	no effect	-	no effect
**Mu50***	0.03125	from 256 to 32	8	-	no effect
	0.0625	from 256 to 8	32	from 32 to 16	2

*MRSA strain; values in bold correspond to a restoration of OXA activity against MRSA.

Table S17. Effect of compound **19** on MICs of OXA and AMP against *S. aureus* strains.

		OXA		AMP	
**Strain**	**Concentration of compound [mM]**	**Numerical value of reduction [µg/ml]**	**Activity gain [A]**	**Numerical value of reduction [µg/ml]**	**Activity gain [A]**
**MM-O058**	0.0625	-	no effect	-	no effect
	0.125	-	no effect	-	no effect
**MM-N072**	0.0625	-	no effect	-	no effect
	0.125	-	no effect	-	no effect
**LG-N017***	0.0625	-	no effect	-	no effect
	0.125	-	no effect	-	no effect
**MM-O021***	0.0625	-	no effect	-	no effect
	0.125	-	no effect	-	no effect
**R45-CC45***	0.0625	-	no effect	-	no effect
	0.125	-	no effect	-	no effect
**R46-CC22***	0.0625	-	no effect	-	no effect
	0.125	-	no effect	-	no effect
**USA300 LAC***	0.0625	-	no effect	-	no effect
	0.125	-	no effect	-	no effect

**5328***	0.0625	-	no effect	-	no effect
	0.125	-	no effect	-	no effect
**COL***	0.0625	-	no effect	-	no effect
	0.125	-	no effect	-	no effect
**Mu50***	0.0625	-	no effect	-	no effect
	0.125	-	no effect	-	no effect

*MRSA strain

Table S18. Effect of compound **20** on MICs of OXA and AMP against *S. aureus* strains.

		**OXA**		**AMP**	
**Strain**	**Concentration of compound [mM]**	**Numerical value of reduction [µg/ml]**	**Activity gain [A]**	**Numerical value of reduction [µg/ml]**	**Activity gain [A]**
**MM-O058**	0.03125	-	no effect	from 8 to 4	2
	0.0625	-	no effect	from 8 to 4	2
**MM-N072**	0.03125	-	no effect	from 32 to 16	2
	0.0625	-	no effect	from 32 to 16	2
**LG-N017***	0.03125	**from 12 to 0.5**	24	from 16 to 8	2
	0.0625	**from 12 to 0.375**	32	from 16 to 6	2.7
**MM-O021***	0.03125	from 48 to 32	1.5	from 128 to 64	2
	0.0625	from 48 to 32	1.5	from 128 to 64	2
**R45-CC45***	0.03125	**from 4 to 0.25**	16	from 48 to 16	3
	0.0625	**from 4 to 0.125**	32	from 48 to 8	6
**R46-CC22***	0.03125	**from 64 to 1**	64	-	no effect
	0.0625	**from 64 to 0.5**	128	-	no effect
**USA300 LAC***	0.03125	**from 12 to 0.5**	24	from 64 to 32	2
	0.0625	**from 12 to 0.375**	32	from 64 to 32	2
**5328***	0.03125	-	no effect	-	no effect
	0.0625	-	no effect	-	no effect
**COL***	0.03125	-	no effect	-	no effect
	0.0625	-	no effect	-	no effect
**Mu50***	0.03125	from 256 to 32	8	-	no effect
	0.0625	from 256 to 8	32	-	no effect

*MRSA strain; values in bold correspond to a restoration of OXA activity against MRSA.

Table S19. Effect of compound **21** on MICs of OXA and AMP against *S. aureus* strains.

		**OXA**		**AMP**	
**Strain**	**Concentration of compound [mM]**	**Numerical value of reduction [µg/ml]**	**Activity gain [A]**	**Numerical value of reduction [µg/ml]**	**Activity gain [A]**
**MM-O058**	0.125	-	no effect	-	no effect
	0.25	-	no effect	-	no effect
**MM-N072**	0.125	-	no effect	-	no effect
	0.25	-	no effect	-	no effect
**LG-N017***	0.125	-	no effect	-	no effect
	0.25	-	no effect	-	no effect
**MM-O021***	0.125	-	no effect	-	no effect
	0.25	-	no effect	-	no effect
**R45-CC45***	0.125	-	no effect	-	no effect
	0.25	-	no effect	-	no effect
**R46-CC22***	0.125	-	no effect	-	no effect
	0.25	-	no effect	-	no effect
**USA300 LAC***	0.125	-	no effect	-	no effect
	0.25	-	no effect	-	no effect

**5328***	0.125	-	no effect	-	no effect
	0.25	-	no effect	-	no effect
**COL***	0.125	-	no effect	-	no effect
	0.25	-	no effect	-	no effect
**Mu50***	0.125	-	no effect	-	no effect
	0.25	-	no effect	-	no effect

*MRSA strain

Table S20. Effect of compound **18** and **20** on MICs of ERY against *S. aureus* strains.

		**Cpd 18**		**Cpd 20**	
**Strain**	**Concentration of compound [mM]**	**Numerical value of reduction [μg/ml]**	**Activity gain [A]**	**Numerical value of reduction [μg/ml]**	**Activity gain [A]**
**MM-O058***	0.03125	-	no effect	-	no effect
	0.0625	-	no effect	-	no effect
**MM-N072***	0.03125	-	no effect	-	no effect
	0.0625	-	no effect	-	no effect
**LG-N017***	0.03125	from 8 to 4	2	**from 8 to 1**	8
	0.0625	from 8 to 4	4	**from 8 to 1**	8
**MM-O021***	0.03125	-	no effect	-	no effect
	0.0625	-	no effect	-	no effect
**R45-CC45**	0.03125	-	no effect	-	no effect
	0.0625	-	no effect	-	no effect
**R46-CC22**	0.03125	-	no effect	-	no effect
	0.0625	-	no effect	-	no effect
**USA300 LAC***	0.03125	from 64 to	3	from 64 to 4	16
	0.0625	from 64 to 12	5.3	from 64 to 2	32
**5328***	0.03125	-	no effect	-	no effect
	0.0625	-	no effect	-	no effect
**COL***	0.03125	-	no effect	-	no effect
	0.0625	-	no effect	-	no effect
**Mu50***	0.03125	-	no effect	-	no effect
	0.0625	-	no effect	-	no effect

Safety studies

AMES test

Table S21. Mutagenic index (**MI**) and other values for doxorubicin (reference compound) and compound **13**.

Compound	Mutagenicity at 1 μM concentration				Mutagenicity at 10 μM concentration			
	MI	Fold increase over baseline	B	SD	MI	Fold increase over baseline	B	SD
DOXO	1.64	1.31	1.00	1.00	0.95	0.77	0.46	3.61
**13**	0.45	0.43	0.01 C	0.58	0.55	0.51	0.01	4.58

(Mutagenic index) MI = mean revertant value for compound tested/mean revertant value for the blind probe with DMSO; compound is considered to be mutagenic if MI ≥ 2; baseline = mean + 1 SD, B – the binomial B value indicates the probability that spontaneous mutation events alone produce at most n = total number of yellow wells at a given concentration of the test sample (3 x 48 wells); a binomial B-value ≥ 0.99 indicates that chances are ≤ 1% that this result occurs due to spontaneous mutation; data points that are significantly smaller (B ≤ 0.01) than the mean number of spontaneous revertants are labelled as C "cytotoxic effect". Compound is mutagenic if data points with fold increase ≥ 2 and Binomial B-value ≥ 0.99.
